# Aberrant Mitochondrial Metabolism in Alzheimer's Disease Links Energy Stress with Ferroptosis

**DOI:** 10.1002/advs.202504175

**Published:** 2025-07-08

**Authors:** Francesca Alves, Darius Lane, Adam Wahida, Md. Jakaria, Pawel Kalinowski, Adam Southon, Abdel Ali Belaidi, Teresa Samperi‐Esteve, Triet Phu Minh Nguyen, Peng Lei, Marcus Krueger, Stefan Mueller, Marcus Conrad, Puja Agarwal, Sue E Leurgans, Julie Schneider, Ashley I. Bush, Scott Ayton

**Affiliations:** ^1^ The Florey Institute of Neuroscience and Mental Health Melbourne 3052 Australia; ^2^ Florey Department of Neuroscience and Mental Health The University of Melbourne Melbourne 3052 Australia; ^3^ Helmholtz Zentrum München Institute of Metabolism and Cell Death 85764 Neuherberg Germany; ^4^ New Therapeutic Targets Group (TargetsLab) Department of Medical Science Faculty of Medicine University of Girona Girona 17003 Spain; ^5^ Department of Neurology and State Key Laboratory of Biotherapy National Clinical Research Center for Geriatrics West China Hospital Sichuan University Chengdu Sichuan 610041 China; ^6^ Institute for Genetics Cologne Excellence Cluster on Cellular Stress Responses in Aging‐Associated Diseases (CECAD) 50931 Cologne Germany; ^7^ Center for Molecular Medicine (CMMC) University of Cologne 50931 Cologne Germany; ^8^ CECAD/CMMC Proteomics Facility Center for Molecular Medicine Cologne (CMMC) University of Cologne 50931 Cologne Germany; ^9^ Translational Redox Biology, Technical University of Munich (TUM) TUM Natural School of Sciences 85748 Garching Germany; ^10^ Rush Alzheimer Disease Center Rush University Medical Center Chicago 60612 USA

**Keywords:** alzheimer's disease, ATP, bioenergetics, ferroptosis, glutathione, mitochondria, neurodegeneration

## Abstract

Alzheimer's disease (AD) is defined by β‐amyloid plaques and tau‐containing neurofibrillary tangles, but the ensuing cellular derangements that culminate in neurodegeneration remain elusive. Here, a mechanistic link between two AD pathophysiological hallmarks: energy insufficiency and oxidative stress is revealed. It is demonstrated that mitochondrial function and glutathione (GSH) flux are coupled, impacting neuronal ferroptosis susceptibility. Analysis of proteomic data from the inferior temporal cortex of 625 subjects along a continuum of clinical and pathological changes in AD, reveals a prominent depletion of mitochondrial proteins. Biogenetic insufficiency in AD is reflected by a concurrent loss of GSH, which requires 2 ATP for its synthesis, and genetic and pharmacologic ATP depletion models confirm that ATP is rate‐limiting for GSH. Accordingly, an unbiased association analysis uncovers mitochondrial proteins in positive correlation with total GSH (t‐GSH) in AD subjects. But mitochondria also consume GSH via the SLC25A39 transporter. It is found that mitochondrial inhibition either increases or decreases ferroptosis susceptibility in cellular models, depending on contextual factors that dictate whether mitochondria act as a net GSH producer or consumer, respectively. Mitochondria therefore control GSH flux, and loss of energy output is consequently demonstrated as a liability for ferroptosis in AD.

## Graphical abstract

Alves et al. reveal how energy loss and oxidative stress, two major features of Alzheimer's disease, are connected. Mitochondria controls the flux of the antioxidant glutathione (GSH), via facilitating both its production and consumption. Energy restriction limits GSH synthesis, conferring vulnerability to cell death by ferroptosis, implicated as a mechanism of neurodegeneration in Alzheimer's disease.

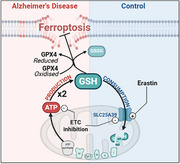

## 1 Introduction

Projections of dementia prevalence are increasing sharply due to demographic change and concurrent increased life expectancy. Alzheimer's disease (AD) is a progressive neurodegenerative disorder and the leading cause of dementia worldwide. It is characterized by cognitive decline and memory loss, and at the molecular level, AD is defined by the accumulation of β‐amyloid plaques and tau neurofibrillary tangles. While these pathological hallmarks have been widely studied, the exact mechanisms driving neurodegeneration and consequent cognitive impairment remain unclear.

So far, therapeutically addressing AD has centered on pathology removal, yet these treatments have yielded only modest clinical benefits with significant risk.^[^ ^1^, ^2^, ^3^ ^]^ One conceptual issue here is that while phenotypic changes are recurrently found, the underlying root cause of neurodegeneration is not yet articulated. Targeting effectors of dysfunction downstream of plaque and tangle pathology may achieve substantial clinical gains for AD patients that are unreached by current therapeutics directed against proteinopathy.

Neuroinflammation, altered mitochondria and energy metabolism, oxidative stress, iron elevation, and protein aggregation have been recurrently implicated in AD,^[^ ^4^ ^]^ but it is unknown which of these critically drive disease, and how they conspire to amplify the cascade of neurodegeneration. Interestingly, these hallmarks share a common association with ferroptosis, an iron dependent cell death pathway that has been recently implicated as a mechanism of neurodegeneration in AD.^[^ ^5^, ^6^, ^7^ ^]^ Ferroptosis is characterized by lipid peroxidation and oxidative damage to cell membranes. Unlike apoptosis or necrosis, ferroptosis can be driven by iron accumulation and the failure of antioxidant defenses, particularly glutathione (GSH) depletion and inactivation of glutathione peroxidase 4 (GPX4). GSH serves as an electron donor for GPX4, allowing it to neutralize reactive oxygen species (ROS) and lipid peroxides.

Hallmarks of ferroptosis have been identified in AD including: reduced glutathione (GSH),^[^ ^8^, ^9^, ^10^, ^11^, ^12^ ^]^ elevated iron,^[^ ^5^, ^13^, ^14^, ^15^ ^]^ and increased lipid peroxidation.^[^ ^16^, ^17^ ^]^ However, a putative physiological trigger for ferroptosis in AD remains unknown. Ferroptosis can be induced in vitro using robust pharmacological inducers such as erastin and RSL3, which respectively indirectly or directly inhibit glutathione peroxidase 4 (GPX4), the master checkpoint for ferroptosis. Erastin inhibits the cystine/glutamate antiporter (system Xc⁻), which supplies cysteine for GSH synthesis, which is consumed by GPX4. Whereas RSL3 directly inhibits GPX4. Although potent ferroptosis inhibitors, such as liproxstatins, have been developed, their clinical translation remains unexplored in part due to the lack of a clearly defined physiological ferroptosis trigger in neurodegenerative diseases. Bridging this gap is crucial for advancing targeted anti‐ferroptotic therapies into clinical trials for AD and related diseases.

In this study, we identify a direct mechanistic link between bioenergetic insufficiency and ferroptosis vulnerability in AD. Through proteomic analysis of 625 post‐mortem brain samples, and subsequent cellular biochemistry work we demonstrate that mitochondrial dysfunction leads to ATP depletion, which in turn limits GSH synthesis, impairing antioxidant defenses. We propose that GSH deficiency in AD is not driven by cysteine depletion or GPX4 dysfunction but is instead directly constrained by ATP availability. We confirm that mitochondrial inhibition exacerbates ferroptosis when ATP is required for GSH production but protects against ferroptosis when mitochondria act as net GSH consumers. Furthermore, we introduce CAP‐17, a bacterial ATP nucleosidase, as a novel tool for selectively depleting ATP and consequently GSH in mammalian cells. These findings establish low ATP as a pathophysiological driver of ferroptosis in AD, highlighting ferroptosis inhibition as a promising therapeutic strategy to slow neurodegeneration.

## 2 Results

### 2.1 Unbiased Proteomic Analysis Reveals Prominent Mitochondrial Protein Loss in AD

**Figure 1:**
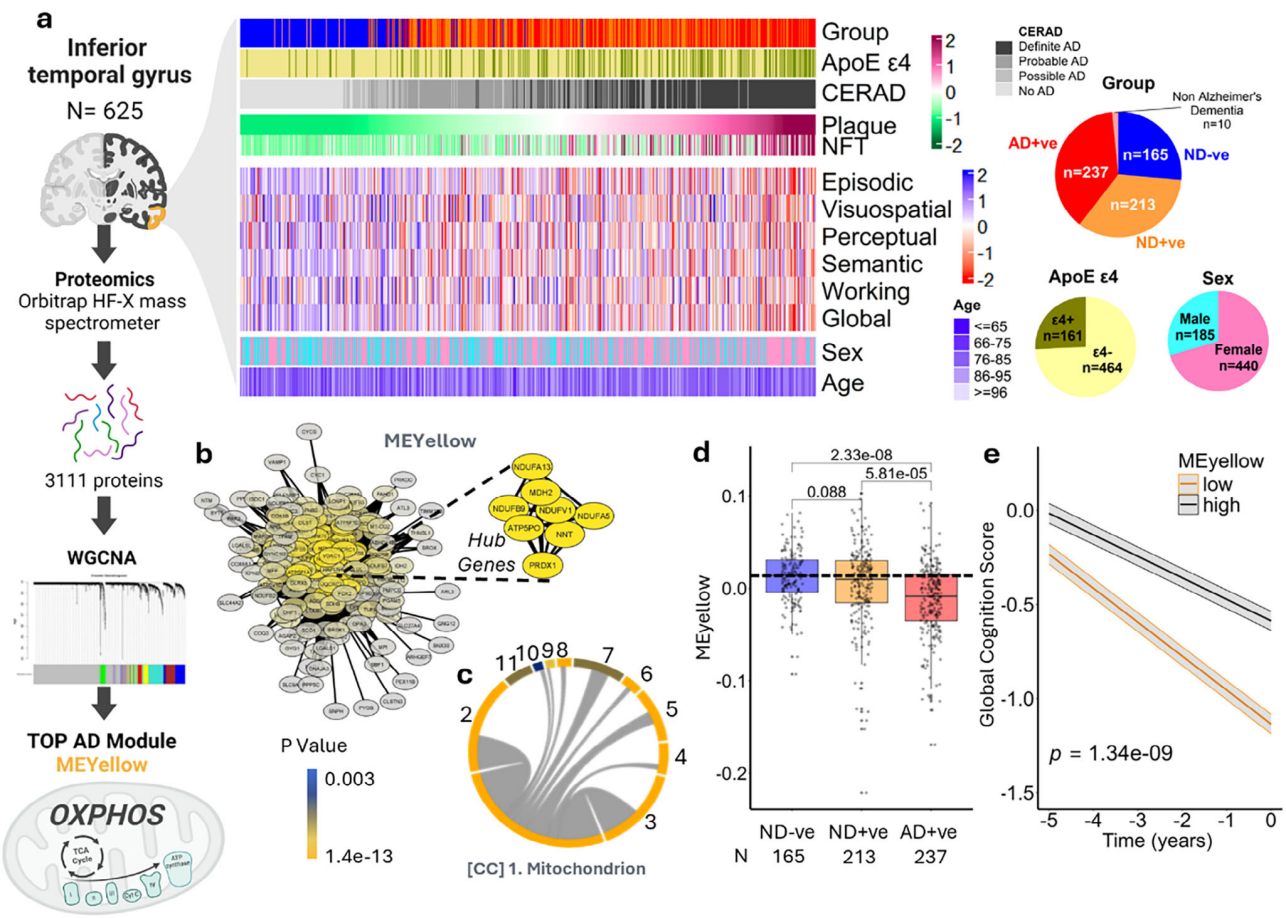
Large proteomic interrogation of inferior temporal gyrus tissue from 625 MAP study participants. a) Cohort metadata overview. The heatmap shows measures of AD pathology, APOE ε4 genotype, cognitive function, sex (rows) for the 625 study participants (columns), ordered by plaque, study cohort composition and proteomics work‐flow including a WGCNA, which identified the yellow module (MEyellow). Pie charts summarise subject grouping by dementia and pathology (based on the CERAD criteria), ApoE ε4 status and sex. b) cytoscape diagram of proteins in the yellow module with highlighted hub genes and c) MonaGO chord plot of cellular component identification of mitochondria as the yellow module; 1. mitochondrion; 2, mitochondrial inner membrane; 3, mitochondrial matrix; 4, respiratory chain; 5, respiratory chain complex I; 6, mitochondrial intermembrane space; 7, membrane raft, intercalated disk; 8, pyruvate dehydrogenase complex; 9, mitochondrial alpha‐ketoglutarate dehydrogenase complex; 10, mitochondrial crista; and 11; postsynaptic density. d) boxplot showing MEyellow eigenvalue score across MAP study participants grouped by dementia and pathology (based on the CERAD criteria) ND‐ve; *n* = 165), people who died without dementia but met criteria for AD pathology (ND +ve; *n* = 213) and people who were diagnosed with AD dementia, and had AD pathology confirmed post mortem (AD+ve *n* = 237); each dot represents an individual subject; one‐way ANOVA. e) global cognition median split high versus low MEyellow over 5 years preceding death (all subjects; *n* = 615). Schematics in a created with BioRender.com.

To interrogate the biochemical sequalae in AD, we conducted proteomic analysis of the inferior temporal gyrus (*n* = 625), utilizing longitudinal cognitive data (*n* = 615) for people who died with: no dementia (ND) without AD pathology (based on the CERAD criteria; ND‐ve; *n* = 165), those who died without dementia but were classified as positive for AD pathology (ND+ve; *n* = 213), and those diagnosed with AD with AD pathology confirmed post mortem (AD+ve *n* = 237; Table S1, Supporting Information). A weighted gene co‐expression network analysis (WGCNA)^[^ ^18^ ^]^ clustered these proteins into modules (Figure S1a–f, Supporting Information); the top module predicting AD with a clear ontology represented oxidative phosphorylation/mitochondria (MEyellow; **Figure** **1**b,c). The AD+ve group was statistically different at baseline from both the ND‐ve (β[95%CI] = 0.025, 0.016, 0.033], *p* = 2.33 × 10^−8^) and ND+ve (β[95%CI] = 0.016, [0.009,0.023], *p* = 5.81 × 10^−5^, Figure 1d) groups. Low oxidative phosphorylation/mitochondria module eigenvalue score was associated with a more rapid cognitive decline in the years prior to death (β time*MEyellow [95%CI] = 0.657 [0.445,0.868], *p* = 1.34 × 10^−9^; high MEyellow, Figure 1e; Figure S1a–e, Supporting Information). Significantly decreased oxidative phosphorylation module scores in AD+ve were confirmed in a validation data set of the dorsolateral prefrontal cortex (Figure S2g–m, Supporting Information, ref^[^ ^19^ ^]^). These findings also are supported by similar proteomics analysis revealing prominent mitochondrial changes in the brains of the AD mouse model, APP CRND8 AD.^[^ ^20^ ^]^

Two questions emerged from finding a clear mitochondrial protein loss in AD: 1. does the reduction in mitochondrial proteins manifest as bioenergetic insufficiency, if so, 2. how does this drive disease progression? We therefore sought additional evidence to substantiate mitochondrial impairment in AD, and its links with neurodegeneration.

### 2.2 Could GSH Report ATP Insufficiency in AD?

**Figure 2:**
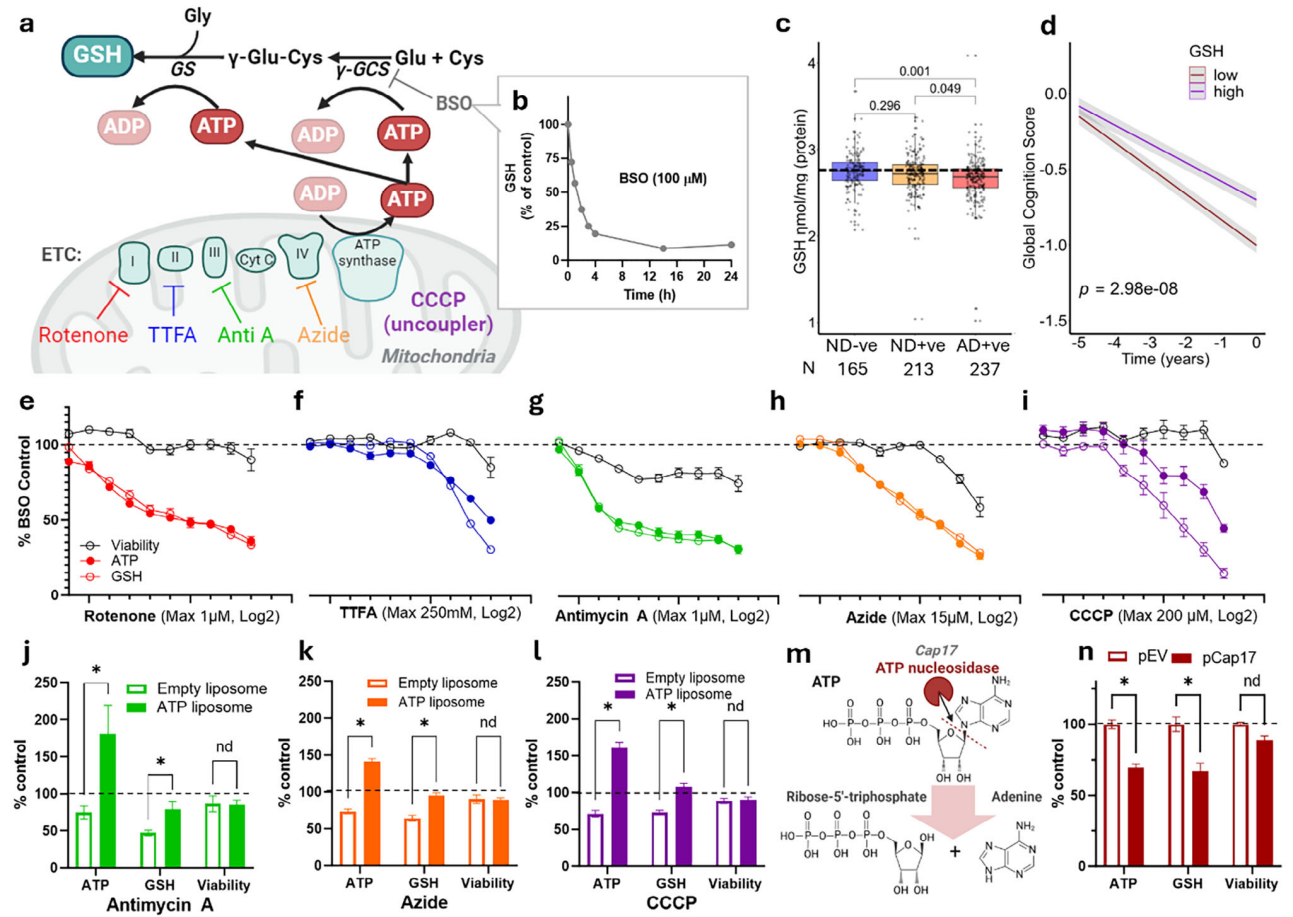
ATP is linked to GSH synthesis revealing ferroptosis vulnerability under energy stress. a) Schematic displaying that two ATP molecules generated by the electron transport chain (ETC) are required to synthesise 1 GSH molecule; γ‐GCS, γ‐glutamylcysteine synthetase; GS, GSH synthetase. b) Time course of t‐GSH depletion with buthionine sulphoximine (BSO) in HT22 neuronal cells. c) boxplot showing t‐GSH across MAP study subjects assigned according to dementia and pathology (CERAD); each dot represents individual subject; a one way ACNOVA with age at death, APOE ε4, and sex as covariates. d) global cognition median split high versus low t‐GSH over 5 years preceding death (all subjects; *n* = 615). e–i) GSH recovery after 4 h of BSO treatment – GSH, ATP and viability were measured in HT22 cells after 17 h of recovery in the presence of escalating doses of ETC inhibitors (rotenone – complex 1 inhibitor; TTFA – complex II inhibitor; antimycin A – Complex III inhibitor; sodium azide – Complex IV inhibitor; CCCP – mitochondrial uncoupler). j–l) Bar plots of ATP, t‐GSH and viability after 17 h co‐treatment with 5% of either ATP‐encapsulated or empty liposomes with ETC inhibitors; antimycin (250 nm), azide (7.5 mm) and CCCP (3.125 µm). m) schematic diagram showing the action of ATP nucleosidase (Cap 17) cleaving the N‐glycosidic bond between the adenine and sugar moieties of ATP, resulting in adenosine and ribulose 5‐triphosphate products. n) bar plots of ATP and t‐GSH in HT22 cells FACS‐sorted for high transfection of Cap17 (pCap17) or empty vector (pEV). Data in j‐l & n presented as mean ± SEM of 3 independent experiments, multiple two‐sided *t*‐tests, ^*^indicates significance *p*<0.05. nd indicates no difference. Schematics in a & m created with BioRender.com.

ATP is the major product of the mitochondria, however, we could not measure ATP because it is not stable in post‐mortem tissues (degraded within 10 min^[^ ^21^ ^]^). Since the production of GSH requires two ATPs (**Figure** **2**a), we reasoned that the abundance of glutathione (GSH) may provide a biochemical indicator of a possible energy insufficiency. GSH is a tripeptide of glutamate, cysteine and glycine that can undergo oxidation and reduction, and is used by glutathione peroxidase 4 (GPX4) to detoxify phospholipid hydroperoxides that drive ferroptosis,^[^ ^22^, ^23^ ^]^ an iron‐dependent cell death pathway implicated in AD.^[^ ^7^, ^24^, ^25^ ^]^ Intracellular GSH is abundant^[^ ^26^ ^]^ (≈5 mm) and is rapidly turned over. Selective blockade of GSH synthesis with buthionine sulphoximine (BSO) causes depletion of t‐GSH within hours (HT22 cells; Figure 2b). The high abundance and fast turnover of t‐GSH necessitates substantial consumption of ATP to maintain the t‐GSH pool.

t‐GSH is reported to be decreased in AD brain,^[^ ^8^, ^10^ ^]^ a change confirmed in our autopsy samples: the AD+ve group had lower t‐GSH compared to the ND‐ve (β[95%CI] = 0.092, [0.041, 0.123], *p* = 0.001) and the ND+ve groups (β[95%CI] = 0.054, 0.009, 0.1], *p* = 0.049. Figure 2c). Low total GSH was associated with accelerated cognitive deterioration (β_time*t‐GSH_[95%CI] = 0.104 [0.067,0.141, *p* = 2.98 × 10^−8^, Figure 2d; Figure S3a–e, Supporting Information). GSH deficiency in AD could be caused by alternative mechanisms (e.g., cysteine limitation). Before exploring other possibilities, we tested whether t‐GSH levels are limited by ATP as we have hypothesized, although to our knowledge, has not previously been demonstrated.

We measured the recovery of t‐GSH levels after BSO‐induced GSH depletion in HT22 cells (with liproxstatin co‐treatment to prevent ferroptosis) in the presence or absence of mitochondrial electron transfer chain (ETC) inhibitors (rotenone – complex 1 inhibitor; TTFA – complex II inhibitor; antimycin A – Complex III inhibitor; sodium azide – Complex IV inhibitor; CCCP – mitochondrial uncoupler) to suppress ATP production. ETC inhibitors induced a striking concordance between the degree of depletion of ATP by mitochondrial inhibitors and the recovery of t‐GSH (Figure 2e–i). Some of these conditions caused a degree of viability loss, which will contribute to the loss of t‐GSH and, likewise, the loss of ATP. While in the above conditions liproxstatin was present to inhibit ferroptosis, we also tested ETC inhibitors without liproxstatin to determine whether these compounds themselves induced ferroptosis, which would confound interpreting the results as our intention here is not to characterise how mitochondrial inhibitors cause cell death. Indeed at these concentrations there could be on target and off target effects of these ETC inhibitors and the data at high concentrations should be interpreted with caution as there was a degree of cell death caused by these drugs. Both rotenone and TTFA caused cell death that was recoverable by liproxstatin (Figure S4a–e, Supporting Information), consistent with previous results,^[^ ^27^, ^28^ ^]^ so we excluded these inhibitors from subsequent experiments. To confirm that the loss of t‐GSH caused by inhibition of the mitochondria was due to ATP insufficiency, we co‐administered ATP‐containing liposomes to HT22 cells under mitochondrial inhibition, which restored t‐GSH levels (Figure 2j–l).

Extant strategies to lower ATP in mammalian cells (i.e., nutrient withdrawal, ETC inhibition, mitochondria uncoupling) all have secondary consequences to metabolism. To directly assess whether lowering ATP could limit t‐GSH levels, we utilized a newly characterized bacterial nucleosidase, Cap17, which selectively degrades cellular ATP (not AMP, ADP, GTP, UTP, CTP) by cleaving the N‐glycosidic bond between the adenine and sugar moieties, resulting in adenine and ribose 5‐triphosphate products (Figure 2m, ref^[^ ^29^ ^]^). We found that the stable expression of nucleosidase domain of Cap17 expression depleted ATP and total glutathione by similar magnitudes (Figure 2n), providing direct evidence that t‐GSH can be limited by ATP.

These data demonstrate that limiting ATP availability also restricts GSH synthesis. To test the reverse possibility—whether inhibiting GSH synthesis might increase ATP levels—we measured ATP following 17 h of BSO treatment, in the presence of LPX to prevent cell death. No change in total ATP was observed (Figure S5, Supporting Information). This is not unexpected, as ATP levels are tightly regulated and typically do not rise inappropriately when consumption decreases. However, this scenario differs from the experimental and disease model we are investigating, in which the cell loses its capacity to supply sufficient ATP to maintain GSH levels—reflecting a failure of normal ATP homeostasis.

### 2.3 Alternative Mechanisms That Regulate GSH Cannot Explain Low t‐GSH in AD

Having demonstrated that low abundance of t‐GSH can be caused by ATP limitation, we next investigated alternative explanations for lower t‐GSH levels in the AD brain. It is possible that t‐GSH is affected by post‐mortem interval. Prior studies report that cortical GSH levels are not impacted by post‐mortem interval,^[^ ^30^ ^]^ which we confirmed in our samples (Figure S6a, Supporting Information).

Oxidative stress is well documented in the AD brain,^[^ ^31^ ^]^ and it is commonly suggested that low GSH levels are a result of increased oxidative stress. GSH becomes oxidised (GSSG) when utilized in the defence against oxidative stress by enzymes such as GPX4, which was unaltered in AD (Figure S6b, Supporting Information), but the t‐GSH (GSH + GSSG) levels remain unchanged. In HT22 cells exposed to high concentrations of the oxidant, menadione, the ratio of GSH to GSSG was suppressed by menadione, but t‐GSH (GSH + GSSG) levels were not altered (Figure S6c, Supporting Information), which is consistent with prior findings.^[^ ^32^ ^]^ In our samples, we used an assay for total GSH + GSSG, not the GSH:GSSG ratio, the latter can be unstable during the agonal period and when the sample is exposed to the atmosphere when preparing samples for analyses. Pro‐oxidant iron may also drive oxidative stress in AD, although iron levels were elevated in AD cases (Figure S6d, Supporting Information) as we previously reported. Iron and total glutathione levels were not associated (Figure S6e, Supporting Information). Negative correlations between t‐GSH and plaque and tangle pathology were observed, but subtle (Figure S6f,g, Supporting Information). Sex (β[95%CI] = −0.049, [−0.091, −0.007], *p* = 0.022), but not *APOE* ε4 risk allele or age at death was associated with t‐GSH levels (Figure S6h,i, Supporting Information).

Cysteine is canonically regarded as rate limiting for GSH synthesis. Indeed, the inhibition of system Xc‐ inhibitors, such as erastin, deplete cysteine levels and induce ferroptosis.^[^ ^23^ ^]^ We measured tissue cystine levels in available data (*n* = 232) and found no difference in AD (Figure S6j, Supporting Information), and a negative association between cysteine and t‐GSH (Figure S6k, Supporting Information) suggesting that cysteine is less efficiently converted into GSH in AD. We measured cystine levels, not cysteine levels, as cysteine is prone to oxidation and was undetectable in a metabolomics analysis. Low t‐GSH was also not explained by changes in the rate‐limiting enzyme of glutathione synthesis, gamma‐glutamylcysteine synthetase (Figure S6l,m, Supporting Information), which was increased in AD+ve. Synthesis enzyme subunit expression may vary according to brain regions, as prior studies have shown a downregulation of Glutamate‐Cysteine Ligase Modifier Subunit (GCLM) in the prefrontal cortex and cerebellum.^[^ ^5^ ^]^

### 2.4 Unbiased Proteome Regression Analysis Confirms t‐GSH Link with Mitochondria is Altered in AD

**Figure 3:**
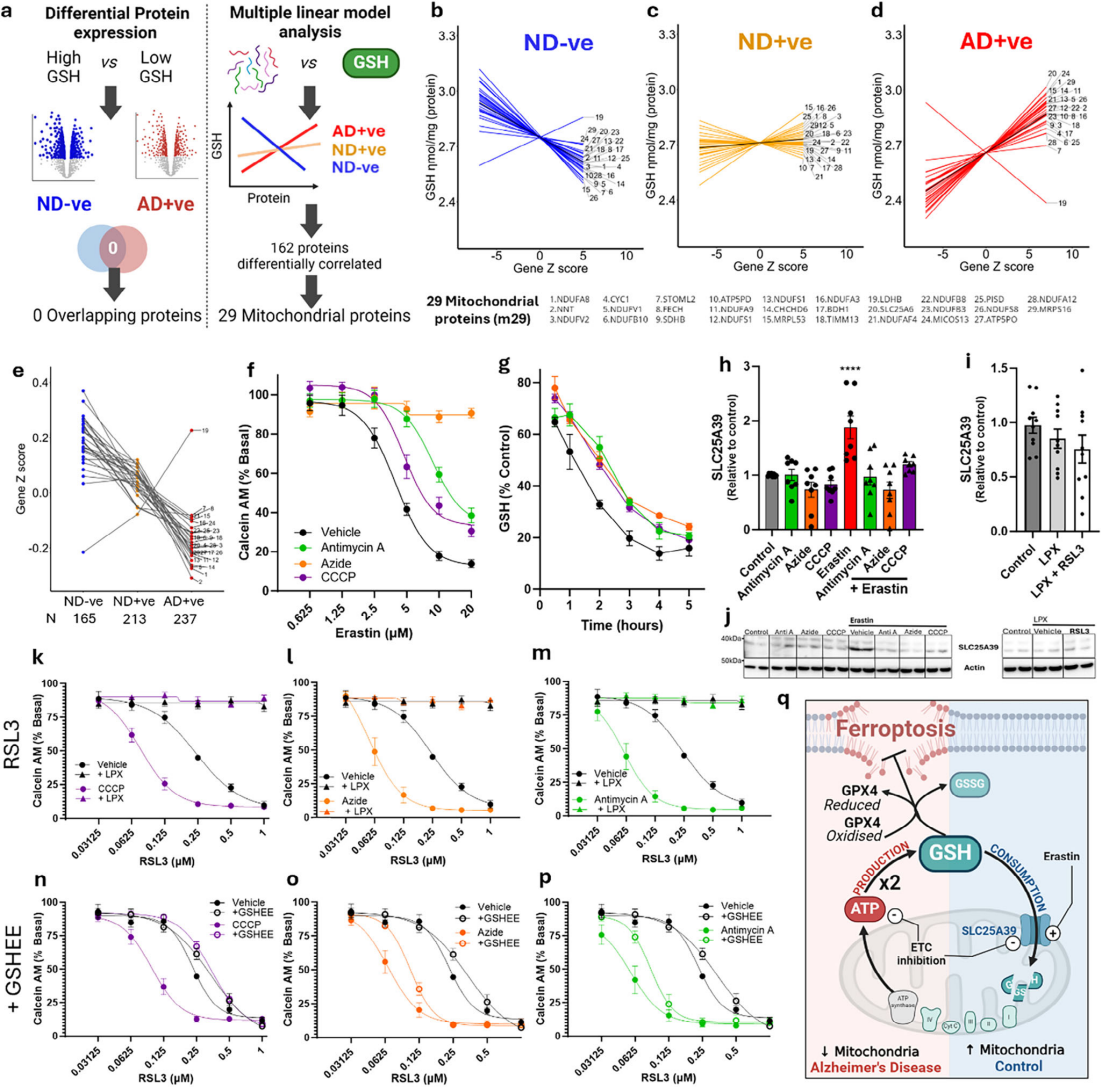
Unbiased analysis of Glutathione in AD reveals a mitochondrial relationship and ferroptosis vulnerability with mitochondrial reduction. a) Workflow schematic of differential protein expression in the MAP proteomics comparing high versus low total GSH, revealing 0 overlap of hits and multiple linear model analysis revealing 162 differentially correlated proteins of which 29 are mitochondrial. b–d) 29 stacked linear models showing the relationship of 29 mitochondrial proteins (Gene Z scores) with total GSH in AD+ve (red, *n* = 237), ND+ve (yellow, *n* = 213), and ND‐ve, pathology‐ve (blue, *n* = 165). e) dot plot of protein expression (Gene Z score) of 29 mitochondrial proteins across MAP subjects assigned according to dementia and pathology (CERAD). f) Viability of HT22 neuronal cells (assayed by Calcein AM) co‐treated with erastin for 17 h in the absence or presence of electron transport chain inhibitors (antimycin A – Complex III inhibitor (green, 200 nm); sodium azide – Complex IV inhibitor (orange, 7.5 mm); CCCP – mitochondrial uncoupler (purple, 3.125 µm)). g) total GSH depletion with erastin (black) in the presence of ETC inhibitors. h) Bar plot of SLC25A39 protein expression (relative to control) in HT22 cells treated in the presence or absence of erastin (10um) with ETC inhibitors, one way ANOVA, ^****^ *p* < 0.0001. i) Bar plot of SLC25A39 protein expression (relative to control) in HT22 cells treated in the presence or absence of RSL3 (1um) + liproxstatin (LPX, 1um) one way ANOVA, ns = not significant. j) Representative western blot images. k–m) viability curves of RSL3 with ETC inhibitors (antimycin A – Complex III inhibitor (green, 200 nm); sodium azide – Complex IV inhibitor (orange, 7.5 mm); CCCP – mitochondrial uncoupler (purple, 3.125 µm) with or without liproxstatin (LPX, 1um) and n–p) with or without glutathione ethyl ester (GSHEE, 1 mm). q, schematic showing the bimodal relationship between mitochondrial protein expression and glutathione created using BioRender.com. k,p data are means± SEM.

To investigate explanations of decreased t‐GSH in AD in an unbiased way, we performed differential co‐expression and Boruta analysis of proteins in ND‐ve and AD+ve subjects stratified by low/high t‐GSH. We found no overlap in proteins corresponding with either high or low total GSH in ND‐ve and AD+ve subjects (**Figure** **3**a; Figure S7a–e, Supporting Information). Having excluded a common driver of total GSH levels in ND‐ve and AD+ve subjects, we investigated protein correlates of total GSH that were directionally opposed in ND‐ve and AD+ve tissue. A series of linear models were run predicting total GSH and the interaction of Group and protein which revealed a clear enrichment of mitochondrial proteins (GO:0005743; 29 mitochondrial proteins; m29). Total GSH was positively associated with mitochondrial protein expression in AD+ve but negatively associated in ND‐ve subjects, while there was a mixed correlation pattern between total GSH and m29 proteins in ND+ve (Figure 3b–d). The one exception to this patten was the protein lactate dehydrogenase B (LDHB), which demonstrated the reverse relationship to other mitochondrial proteins. While LDHB has been found in the mitochondria, it is predominantly cytosolic.^[^ ^33^ ^]^

In AD subjects, the direction of the relationship between total GSH and m29 proteins supports the possibility of ATP being a limiting factor for GSH synthesis, since lower mitochondrial protein expression was associated with lower total GSH levels. Again with the exception of LDHB, the total levels of m29 group of proteins were decreased in AD+ve (Figure 3e), consistent with our findings in the MEyellow module (Figure 1c) and a recent compilation of proteomic studies in AD^[^ ^34^ ^]^ that predicts a deficiency of mitochondrial proteins required to generate ATP for GSH production. These findings agree with our cellular experiments that demonstrate that ATP insufficiency can limit GSH to a similar proportion. Having explored other alternative explanations in the AD tissue, this unbiased analysis supports our hypothesis that low GSH in AD is driven by low ATP, but we cannot exclude other potential unknown causes of low GSH, including increased GSH consumption. However, in ND‐ve subjects there was negative relationship between m29 and total GSH; opposite to what was observed in AD+ve subjects. This negative relationship implies ATP is not limiting for GSH synthesis in ND‐ve subjects where the mitochondrial proteins are adequate, but is limiting in AD.

### 2.5 Mitochondrial Inhibition Prevents GSH Consumption by the Mitochondria

When investigating why low mitochondrial proteins would be correlated with higher total GSH in ND‐ve subjects, we recalled a paradigm in ferroptosis that is consistent with this relationship. Mitochondrial inhibitors protect against cysteine deficiency‐induced ferroptosis, which causes cell death by t‐GSH depletion. These studies proposed that erastin‐induced depletion of t‐GSH was attenuated by mitochondrial inhibition due to a lower oxidative stress burden, since mitochondrial activity produces oxidative stress. Consistent with this, we found that ETC inhibitors provide protection against ferroptosis induced by cysteine deficiency by erastin (Figure 3f). Of note, azide clearly demonstrates the greatest protection, and this may be related to other effects that go beyond the preservation of GSH, which is the focus of the current study.

While there may be additional mechanisms contributing to the protection against ferroptosis conferred by decreased oxidative phosphorylation, our findings support the proposed GSH preservation mechanism by showing that ETC inhibitors blunt the reduction of total GSH by erastin (Figure 3g). However, maintenance of t‐GSH caused by ETC inhibition in cysteine deficiency‐induced ferroptosis is not due to lower oxidative stress, since neither the mitochondrial‐specific antioxidant, mitotempo, nor the anti‐ferroptosis agent, liproxstatin, similarly preserves t‐GSH levels (Figure S8a, Supporting Information), and mitochondrial ROS are shown not to be elevated under similar conditions.^[^ ^23^ ^]^

### 2.6 Mitochondrial Inhibition Prevents t‐GSH Consumption Via SLC25A39

Recently, it was reported that mitochondria consume GSH via the transporter SLC25A39.^[^ ^35^, ^36^, ^37^, ^38^ ^]^ SLC25A39 is elevated in conditions of GSH deficiency, for example by erastin treatment, revealing the biased preservation of mitochondrial GSH at the expense of cytosolic GSH. GSH uptake by the mitochondria in cysteine deficient conditions is a strategy to deliver sulfur for iron‐sulfur cluster biogenesis that preserves OXPHOS capacity and ATP production.^[^ ^39^ ^]^ We confirmed that SLC25A39 levels increase with erastin exposure in HT22 cells and demonstrate that this elevation is abolished by mitochondrial inhibitors, explaining why t‐GSH levels are preserved by mitochondrial inhibitors with erastin (Figure 3h,j). Indeed, when we used a paradigm to deplete ATP and GSH depletion with ETC pre‐treatment (as previously shown in Figure S8c–e, Supporting Information), then washed of the ETC inhibitors to permit mitochondrial GSH consumption, ETC pretreated cells were more vulnerable to erastin (Figure S9a, Supporting Information). It would be interesting to test whether mitochondrial inhibition still preserves GSH levels in erastin‐treated cells with SLC25A39 ablated, but knockout of this gene sensitises cells to cuproptosis, which complicates the experiment.^[^ ^40^ ^]^ Regardless, the directionally opposite effects of mitochondrial inhibition on GSH preservation and ferroptosis sensitisation in erastin and RSL3 treated cells align with the observation that former induces SLC25A39 elevation and the latter does not.

We were not able to investigate SLC25A39 in our human samples. Membrane proteins, like SLC25A39, are difficult to detect with mass‐spectrometry (Orbitrap HF‐X mass spectrometer), and indeed, we were unable to detect this protein in our proteomic analysis. Still, mitochondria ND‐ve subjects have markedly greater mitochondrial proteins compared to AD, and it is expected that SLC25A3 would likewise be higher in ND‐ve compared to AD). The negative association between mitochondrial proteins and GSH highlight the hunger of the mitochondria for GSH. When mitochondria act as a net consumer of GSH, such as when we modeled with erastin that induces consumption via SLC25A39, mitochondrial inhibitors prevent against erastin induced ferroptosis, prevent elevation of SLC25A39 and preserve of t‐GSH (Figure 3q).

### 2.7 Mitochondrial Inhibition Lowers ATP‐Dependent GSH Synthesis

We have also shown that mitochondria promote GSH production through the supply of ATP. We hypothesized that when the net effect of mitochondria is to synthesise GSH via ATP (as opposed to a net consumer of GSH via SLC25A39), mitochondrial inhibition would confer susceptibility to ferroptosis. To model this paradigm, we used RSL3 as a ferroptosis inducer that does not cause increased GSH consumption by the mitochondria via the elevation of SLC25A39^[^ ^38^ ^]^ (Figure 3i). Here, mitochondrial inhibitors that lower ATP and t‐GSH over 5 h (Figure S8c–e, Supporting Information) and 17 h (Figure S8f–h, Supporting Information) dramatically exacerbate toxicity to RSL3, which was rescued by liproxstatin (Figure 3k–m), as previously shown,^[^ ^41^ ^]^ but not by the mitochondrial antioxidant, mitotempo (Figure S8i–k, Supporting Information). It is possible that ETC inhibition will deprive ATP from other processes (i.e,. transcription, translation and enzyme activity) that may also contribute to increased ferroptosis vulnerability, however to demonstrate that a material amount of exacerbation of RSL3‐induced ferroptosis is caused by t‐GSH depletion, we supplemented these cells with the GSH donor, GSHEE, and found that this attenuated the increased vulnerability to RSL3 that was induced by the mitochondrial inhibitors (Figure 3n–p).

It is also possible that mitochondrial inhibitors could induce mitophagy, that would also contribute to the observed preservation of GSH. Therefore, to compare the impact of altered mitochondrial function without relying on ETC inhibitors, we investigated the impact of ferroptosis on Rho nought (ρ⁰) cells, which lack functional mitochondrial. ρ⁰ cells exhibited a reduction in ATP compared to WT controls, which coincided with low GSH (Figure S10a, Supporting Information). This is as expected, since our prior experiments showed that lowering ATP depletes GSH. Accordingly, this made ρ⁰ cells more vulnerable to ferroptosis induced by RSL3 (Figure S10c, Supporting Information). In contrast, ρ⁰ cells were protected against erastin induced ferroptosis (Figure S10d, Supporting Information). In this case, it is ATP and not cystine that is limiting for GSH synthesis in ρ⁰ cells, so the impact on erastin (which causes cystine limitation) has less of an effect. Furthermore, the mitochondria are not acting as consumers of GSH in ρ⁰ cells, therefore preserving the remaining GSH in the cell when erastin is applied. Therefore, there was mitigation of GSH loss in ρ⁰ cells in response to erastin.

### 2.8 Modeling Ferroptosis Vulnerability in Human Cohort Reveals Increased Prevalence of AD in People With Low Mitochondria and High Iron Levels

The above cellular work employed erastin and RSL3 as tools to probe ferroptosis signaling, but these might not be relevant to AD, since GPX4 and cystine are not deficient. Similarly, SLC25A39 was investigated to explain the opposing results of mitochondrial inhibitors on erastin and RSL3. These tools and models help to understand ferroptosis biology. For example, the concept that loss of mitochondria can limit the consumption of GSH, a mechanism which was gleaned from reducing mitochondrial GSH import via SLC25A39 using mitochondrial inhibitors, explains the negative correlation between mitochondria and GSH seen in ND‐ve (Figure 3b). The same mechanism explains why mitochondrial inhibitors preserve GSH in response to erastin (and also protect against erastin‐induced ferroptosis).

Conversely, we showed that the mitochondria contribute to the synthesis and production of GSH via supplying ATP. This is relevant to AD, where mitochondria and GSH are both decreased, and the positive correlation between GSH and mitochondrial proteins aligns with the concept that mitochondria output ATP that sustains the GSH pool. The same concept of mitochondrial‐dependent GSH production (via ATP) is modeled using chemical treatments such as iron and RSL3, which, unlike erastin, do not stimulate GSH consumption via mitochondrial SLC25A39 induction. In this condition (i.e., iron or RSL3), mitochondrial inhibition has the reverse on ferroptosis sensitization compared to erastin. This paradox is explained by the different effects erastin and RSL3 have of mitochondrial GSH consumption.

**Figure 4:**
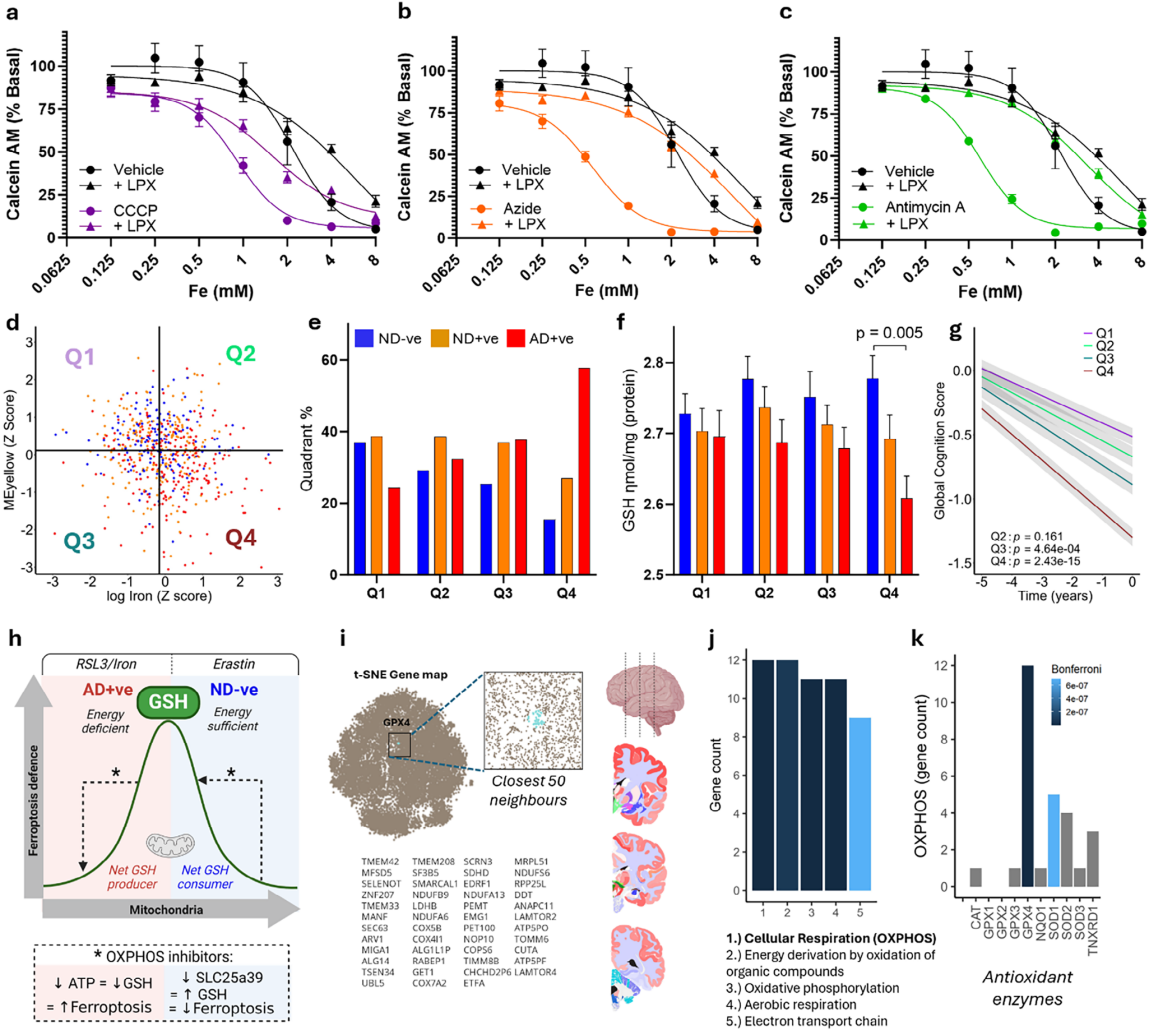
Low mitochondria and high iron models an increased ferroptosis risk present in the AD population. a–c) viability curves cells treated with arachidonic acid and increasing doses of iron in the absence and presence of ETC inhibitors (antimycin A – Complex III inhibitor (green, dose); sodium azide – Complex IV inhibitor (orange, dose); CCCP – mitochondrial uncoupler (purple, dose), and with or without liproxstatin (lpx, 1um) d) Quadrant scatter plot using median values of Mitochondria (MEyellow) and iron from MAP subjects: high mitochondria, low iron (Q1), high mitochondria, high iron (Q2), low mitochondria, low iron (Q3) and low mitochondria, high iron (Q4). Each dot represents a participant (AD+ve = red, ND+ve = yellow, ND‐ve = blue). e) Bar plot of percentage of subjects per quadrant according to diagnosis and pathology status. f) Bar plot of total GSH of subjects per quadrant according to diagnosis and pathology status; p values generated using planned comparisons from a one way ANOVA g) global cognition (95%CI) according to quadrant assignment over 5 years preceding death. h) schematic depicting bimodal relationship between mitochondria as GSH consumer and producer i) t‐SNE Gene map from brainscope.nl; an online interface compiling the co‐expression of transcripts across numerous subjects and brain regions using the Alan Brain atlas highlighting glutathione peroxidase 4 (GPX4) in blue with the top 50 co‐expressed genes. j) Bar plot of gene count from pathway enrichment of the top 50 genes clustered with GPX4. k) Bar plot of oxidative phosphorylation gene count from pathway enrichment of antioxidant enzymes (Catalase, CAT; glutathione peroxidase 1, GPX1; glutathione peroxidase 2, GPX2; glutathione peroxidase 3, GPX3; glutathione peroxidase 4, GPX4; NAD(P)H quinone dehydrogenase 1, NQO1; Superoxide dismutase 1, SOD1; Superoxide dismutase 2, SOD2; Superoxide dismutase 3, SOD3; thioredoxin reductase 1, TNXRD1.

Since erastin and RSL3 are not physiologically relevant inducers of ferroptosis, we examined pathophysiological ferroptosis stressors – iron and arachidonic acid – to investigate what effect mitochondrial inhibitors had on their toxicity. Mitochondrial inhibitors caused vulnerability toward arachidonic acid and iron that was recoverable by liproxstatin (**Figure** **4**a–c). Thus, increased vulnerability to ferroptosis caused by iron and arachidonic acid occurs when mitochondria are impaired. Levels of these stressors that are safe when mitochondria are fully functional are toxic when mitochondria function is low.

To apply this rationale to a clinical context, we investigated the profile of subjects in our tissue cohort with high iron and low mitochondrial proteins. The MEyellow (OXPHOS module) had a modest but statistically significant association with iron (r^2^ = 0.02; *p* = 0.0001; Figure 4d). So, using median values of OXPHOS module and iron, we stratified the cohort into quadrants of: high mitochondrial protein, low iron (Q1), high mitochondrial protein, high iron (Q2), low mitochondrial protein, low iron (Q3) and low mitochondrial protein, high iron (Q4). We found an increased proportion of AD+ve subjects, and a marked decrease of ND‐ve subjects in the quadrant with low mitochondrial proteins and high iron (Q4) (Figure 4e).

Yet, there were still ND‐ve subjects who fit in the Q4 risk quadrant. Our prior analysis revealed that the relationship between mitochondrial protein levels and t‐GSH in ND‐ve is opposite to that of AD+ve (Figure 3a–e); low mitochondrial protein levels might be associated with a protective increase in t‐GSH in ND‐ve but are associated with lower t‐GSH in AD+ve. ND‐ve within the low mitochondrial protein quadrants (Q3 & 4) had elevated t‐GSH compared to AD+ve subjects (β[95%CI] = 0.161[−0.264, −0.059], *p* = 0.0046) (Figure 4f). Subjects in quadrant 4 also had a conspicuous acceleration of cognitive decline (β[95%CI] = −0.09[−0.114, −0.069], *p* = 2.430^*^10^−15^) (Figure 4g; Figure S11a–e, Supporting Information). Low energy leading to low t‐GSH could therefore be a pathophysiological driver of ferroptosis in AD and many other diseases.

Experimental ferroptosis is classically induced by cyst(e)ine depletion (leading to GSH loss) or direct GPX4 inhibition, but we found that this does not occur in AD, and it is unclear if clinically meaningful cysteine deficiency or GPX4 impairment are important for any common chronic disease. This gap between experimental models of ferroptosis and pathophysiology of chronic disease has impaired clinical translation of ferroptosis frameworks into disease‐relevant contexts. Filling this void, we reveal that sufficient ATP is critical to maintain the pool of GSH, and we implicate low cellular energy as a liability for ferroptosis, despite unchanged levels of cysteine and GPX4.

However, the role of mitochondria in ferroptosis is context dependent – we show that when ATP is not limiting for GSH maintenance, mitochondria are net GSH consumers (to maintain iron‐Sulphur biogenesis^[^ ^39^ ^]^) and confer susceptibility to ferroptosis. The role of mitochondria in controlling GSH flux through both production and consumption explains the present and prior findings showing that mitochondrial inhibition protects against cysteine‐deficiency induced ferroptosis, but sensitizes to other ferroptotic cues (Figure 4h, Ref^[^ ^41^, ^42^ ^]^). Similarly, AMPK, which is activated upon acute energy depletion, can protect or sensitize to ferroptosis, depending on context.^[^ ^43^, ^44^, ^45^, ^46^ ^]^

### 2.9 GSH Provides a Fundamental Link Between Energy Production and Protection Against Reactive Oxygen Species Produced by Mitochondria

Beyond AD, GSH synthesis requires ATP across all kingdoms of life, however, GSH is only present in organisms that partake in aerobic metabolism.^[^ ^47^ ^]^ Almost all eukaryotes utilize GSH, except those few that lack mitochondria, such as *entamoeba histolyti*ca.^[^ ^48^ ^]^ The emergence of abundant atmospheric oxygen 2.5 billion years ago revolutionized cellular manufacturing of ATP via oxidative phosphorylation, however this came at a cost of amplified oxidative stress and increased breakdown of Fe‐S clusters. GSH presents as a solution for both by combating oxidative stress, and as a sulphur source for the mitochondria to produce Fe‐S clusters. Mitochondria are responsible for the production of the majority of reactive oxygen species (ROS),^[^ ^49^ ^]^ and they synthesize Fe‐S clusters, requiring abundant GSH. Elevated oxygen stimulates Fe‐S cluster synthesis, and if this is inhibited, OXPHOS is impaired and an iron‐starvation response results in the accumulation of iron and ferroptosis sensitization,^[^ ^50^ ^]^ reminiscent of our findings in AD. Our findings that sufficient ATP is critical to maintain the GSH pool intuitively couples the mitochondrial production of ATP with the defense against ferroptosis.

To validate that this relationship is pertinent to the brain, we used the Brainscope Atlas database to profile genes that are spatially co‐expressed with GPX4 across the human brain (Figure 4i). Gene ontology enrichment of the top 50 genes revealed the highest ranked biological process associated with GPX4 was oxidative phosphorylation (Figure 4j). This relationship of an antioxidant protein with oxidative phosphorylation may be expected since mitochondria are substantial generators of reactive oxygen species, but GPX4 stands out compared to other key antioxidant genes that were not as associated with oxidative phosphorylation gene expression (Figure 4k). The decoupling of this ancient relationship of mitochondria with GSH flux in AD may therefore erode the defense against ferroptotic neurodegeneration. In AD, where low mitochondrial proteins accompany low t‐GSH, drugs that inhibit ferroptosis may have the dual benefit of directly bolstering neurons with weakened ferroptosis defenses, as well as reducing the tax on the already depleted ATP pool in the supply of sufficient GSH.

## 3 Conclusion

This study reveals a new mechanistic link between two well‐established pathophysiological changes in AD, energy insufficiency and oxidative stress, and exposes a druggable pathway to slow neurodegeneration. Low energy leading to low GSH could be a pathophysiological driver of ferroptosis in AD and a host of other neurodegenerative diseases characterized by mitochondrial impairment. Since lowering iron directly using deferiprone led to unfavorable outcomes in AD patients (possibly via starving the mitochondria of iron),^[^ ^51^ ^]^ drugs that instead improve metabolic flux and energy production could be therapeutic for AD. However, this is a challenging – simply increasing nutrient availability will not necessarily lead to increased energy production. Approaches such as mitochondrial transplantation or agents that confer mitochondrial protection such as mitoQ, warrant further investigation for AD.^[^ ^52^, ^53^ ^]^ In addition, treating ferroptosis may have the dual benefit of protecting the damaged membrane of a neuron, while also reducing the tax on the ATP pool for other vital functions impaired in AD (**Table** **1**).

**Table 1:** Baseline demographics.

Variable	Overall	No Dementia ‐ve	No Dementia +ve	AD+	p value
n	626	165	213	237	
Age (Death) mean (SD)	90.16 (6.25)	88.11 (6.60)	90.29 (6.30)	91.43 (5.58)	7.46E‐07
Education mean (SD)	14.60 (2.88)	15.03 (2.71)	14.49 (2.88)	14.42 (2.99)	0.088
Sex (Male) n (%)	0.30 (0.46)	0.34 (0.47)	0.28 (0.45)	0.27 (0.45)	0.328
*APOE* ε4 (Positive) n (%)	161 (25.7)	16 (9.7)	49 (23.0)	94 (39.7)	6.40E‐11
Age (Baseline) mean (SD)	82.80 (5.95)	81.87 (6.23)	82.38 (6.17)	83.83 (5.35)	0.002
Plaque mean (SD)	0.93 (0.84)	0.07 (0.15)	1.10 (0.63)	1.41 (0.82)	2.92E‐75
Neurofibrillary tangles mean (SD)	0.72 (0.81)	0.25 (0.26)	0.64 (0.64)	1.13 (0.98)	2.37E‐29
Episodic memory mean (SD)	−0.20 (0.86)	0.20 (0.59)	0.03 (0.62)	−0.70 (0.99)	3.36E‐31
Visuospatial ability mean (SD)	−0.05 (0.81)	0.16 (0.74)	0.03 (0.80)	−0.26 (0.82)	1.13E‐06
Perceptual Speed mean (SD)	−0.17 (0.82)	0.10 (0.80)	−0.03 (0.70)	−0.48 (0.83)	1.30E‐13
Semantic memory mean (SD)	−0.07 (0.71)	0.20 (0.47)	0.08 (0.54)	−0.41 (0.86)	2.62E‐20
Working memory mean (SD)	−0.05 (0.78)	0.16 (0.75)	0.10 (0.73)	−0.33 (0.78)	1.76E‐11
Global Composite mean (SD)	−0.14 (0.66)	0.17 (0.46)	0.04 (0.47)	−0.53 (0.76)	4.83E‐32
Post mortem interval (SD)	8.03 (4.60)	7.89 (4.03)	8.53 (5.17)	7.76 (4.41)	0.127

## 4 Experimental Section

### The Memory and Ageing Project (MAP)

The Memory and Aging Project (MAP) was an ongoing clinical‐neuropathological cohort study of older adults that began in 1997 and includes residents of retirement communities and subsidized housing in Chicago.^[^ ^54^ ^]^ All participants enrolled without known dementia and agreed to detailed clinical evaluation and brain donation at death.^[^ ^55^ ^]^ Studies were approved by an Institutional Review Board of Rush University Medical Center (ROS IRB# L91020181, MAP IRB# L86121802). Studies were conducted according to the principles expressed in the Declaration of Helsinki. Each participant signed an informed consent, Anatomic Gift Act, and an RADC Repository consent (IRB# L99032481) allowing their data and biospecimens to be repurposed. From the beginning of the MAP study in 1997 until 2017, 884 MAP subjects died and had an autopsy. Out of these 680 subjects, a subset used for this study (*n* = 625) had available neuropathological data, cognitive data, and brain metals a previously reported.^[^ ^56^ ^]^

### Clinical Evaluation Procedures

All subjects underwent a uniform, structured, clinical evaluation that included self‐reported medical history, a neurologic examination by a nurse, and cognitive testing by a neuropsychological test technician, as previously described.^[^ ^57^ ^]^ Years of formal education, and history of change in memory and other cognitive abilities relative to 5 years earlier were documented. All medications used in the prior two weeks were directly inspected and recorded. A complete neurologic examination was performed by nurses, who documented evidence of stroke or Parkinsonian signs. AD clinical diagnosis was based on the Consortium to Establish a Registry for Alzheimer's Disease [CERAD].

### Cognitive Tests

Cognitive performance tests have previously been described for this cohort.^[^ ^57^ ^]^ A battery of 19 cognitive tests, including tests of the Consortium to Establish a Registry for Alzheimer Disease (CERAD), are administered annually to assess a broad range of cognitive abilities that appear to have different anatomic substrates commonly affected by AD and/or widely used for clinical classification of dementia. The battery includes multiple tests of each of 5 cognitive domains: 7 tests of episodic memory (Word List Memory, Word List Recall and Word List Recognition from the procedures established by the CERAD; immediate and delayed recall of Story A from the Logical Memory subtest of the Wechsler Memory Scale‐Revised; and immediate and delayed recall of the East Boston Story), 3 tests of semantic memory (15‐item Boston Naming Test, Verbal Fluency, 15‐item word reading test), 3 tests of working memory (Digit Span Forward, Digit Span Backward, Digit Ordering), 4 tests of perceptual speed (symbol digits modality, Stroop color naming, Stroop word reading, number comparisons) and 2 tests of visuospatial ability (Judgement of Line Orientation and 16‐item version of the Standard Progressive Matrices). Composite scores were computed for each of the cognitive domains and for global cognition by correcting for direction for decline, converting raw scores on each test to standardized scores using the mean and standard deviation from all baseline MAP evaluations, and then averaging the standardized scores to yield the composite z‐scores. Summary measures have the advantage of minimizing floor and ceiling effects, and other sources of random variability.

### Brain Neuropathology

The methods for brain autopsies and pathologic evaluations are described in detail elsewhere.^[^ ^57^ ^]^ A board‐certified neuropathologist, blinded to participant ages and clinical data, determined the neuropathology diagnoses. Briefly, slabs from 1 cerebral hemisphere were placed in a −80 °C freezer and used for metal analyses. Slabs from the contralateral hemisphere were fixed in 4% paraformaldehyde, and then dissected tissue samples from brain regions were embedded in paraffin blocks, cut into 6‐micron sections, and mounted onto slides.

### Alzheimer Disease Neuropathologies

Diffuse and neuritic amyloid plaques and neurofibrillary tangles were identified using modified Bielschowsky silver‐stained 6‐micron sections in multiple cortical regions. Raw counts (greatest density in 1‐mm^2^ area) of the neuritic and diffuse plaques and tangles were standardized in each region (entorhinal cortex, hippocampus, mid‐temporal cortex, inferior parietal cortex, mid‐frontal cortex) and averaged across regions as summary scores that reflect a global measure of Alzheimer disease pathology. Neuropathologic data was categorized based on: CERAD (Consortium to Establish a Registry for Alzheimer's Disease) criteria assess the likelihood of AD pathological diagnosis using the extent of plaque pathology and assigns either rating of possible, probable or definite.

### Brain Tissue Preparation for Proteomics

Inferior temporal gyrus brain tissue (≈50 mg each) was divided into two equal pieces. One was immediately returned to −80 °C, the other was used for protein digestion. All steps were carried out in a dish placed on a dry ice bed using pre‐chilled tools and vials in order to prevent thawing. The material used for protein digestion was immediately transferred to pre‐chilled 2 mL screw cap vials, weighed on an analytical balance and returned to storage at −80 °C until further processing. Individual weights ranged from 16 to 30 mg.

### Tissue Lysis and Digestion

250 µL of cooled lysis buffer (6 m Guanidinium hydrochloride in 50 mm TEAB pH 8.5, 5 mm TCEP, 10 mm CAA, 1 x Halt Protease & Phosphatase Inhibitor Cocktail, Thermo) and 900 mg ceramic beads (6:4 mixture of 1.4 and 2.8 mm diameters, Bertin) were added to each sample and the tissue was homogenized using a precooled Precellys (Bertin) with two cycles of 20 sec at 5800 rpm. After lysis the tubes were centrifuged for 5 min at 20.000 x g, 4 °C. 200 µL supernatant were transferred to fresh tubes, heated to 95 °C for 10 min and sonicated using a Bioruptor (Diagenode) with 10 × 30 sec pulses and 30 sec delay between pulses. 25 µg LysC (Wako) were added to the lysed samples and after incubation for 2 h at 37 °C, 500 µL of 50 mm TEAB pH 8.5 followed by 25 µg Trypsin were added and proteins were digested for 16 h at 37 °C. Digestion was stopped by acidification with 80 µL 10% TFA and the samples were centrifuged for 10 min at 20 000 x g, 4 °C.

### Clean‐Up

96 well C‐18 extraction plates (Sep‐Pack C18, 40 mg, Waters) were activated with 1 × 500 µL methanol followed by 1 × 500 µL 60% CAN / 0.1% TFA using a vacuum manifold with the lowest flow settings. Subsequently the plates were equilibrated with 2 × 1000 µL 0.1% TFA and the acidified digests were loaded onto the plates. The resin was washed with 2 × 1000 µL 0.1% TFA and the peptides were eluted into a receiver plate with 1 × 1000 µL 60% CAN in 0.1% TFA. For proteome measurements 50 µL of the eluate were transferred to 96 well PCR plates, dried in a vacuum centrifuge and resuspended in 100 µl 5% FA, 2% CAN and. 2 × 450 µL µL were transferred to 96 DWP, dried in a vacuum centrifuge and stored at −80 °C for optional analysis of enriched 12 hosphor peptides.

### Proteomics Data Acquisition—Gas Phase Fractionation DIA for Library Generation

Aliquots of 96 randomly selected samples were pooled and used for spectrum library generation by narrow window DIA of six 100 m/z gas phase fractions (GPF) covering the range from 400 to 1000 m/z.^[^ ^58^ ^]^ LC MS analysis of the sample pools was performed on a Orbitrap HF‐X mass spectrometer coupled to an easy LC 1200 nano HPLC system (Thermo) using a pulled tip 75 µm x 500 mm column packed in house with Poroshell C18 2,7 (Agilent). The column was placed in a column oven (Sonation) and operated at 50 °C with a flow rate of 250 nL min^−1^. After pre ‐equilibration in buffer A 0.1% FA) 3 µL of the pool were injected and peptides were separated using a gradient of 4% to 25% buffer B (0.1% FA in 80% ACN) in 96 min followed by 25% to 55% in 14 min, 55% to 95% in 2 and 8 min final column regeneration at 95%. The Orbitrap was operated in DIA mode. MS1 scans of the respective 100 m/z gas phase fraction were acquired at 30k resolution. Maximum injection time was set to 50 msec and the AGC target to 3E6. DIA scans of the corresponding 100 m/z region were acquired in 25 × 4 m/z staggered windows resulting in nominal 2 m/z windows after demultiplexing. MS2 settings were 15 k resolution, a maximum injection time 55 msec and an AGC target of 1e6. All scans were stored as centroid.

### LC MS Analysis

Sample aliquots were diluted 1:10 with 0.1% TFA and 20 µL were loaded onto EvoTips according to the protocol recommended by the manufacturer (EvoSep, https://www.evosep.com/evotip/). LC MS analysis was performed on a Orbitrap HF‐X mass spectrometer coupled to an EvoSep One nano HPLC system^[^ ^59^ ^]^ using a pulled tip 150 µm x 150 mm column packed in house with Poroshell C‐18 2,7 (Agilent). The column was placed in a column oven at 50 °C (Sonation). Samples were eluted from the Evotips onto the column and peptides were separated using the optimized Evosep 30SPD method providing fast 44 min gradients. The Orbitrap HF‐X mass spectrometer (Thermo) was operated in DIA mode. MS1 scans were acquired from 390 to 890 m/z at 30k. Maximum injection time was set to 25 msec and the AGC target to 3E6. MS2 scans were acquired at 15 k resolution with a maximum injection time of 22 msec and an AGC target of 1e6. 40 × 12 m/z staggered windows covered the precursor range from 400 to 880 m/z. All scans were stored as centroid.

### Data Processing

Preprocessing: Thermo raw files were demultiplexed and transformed to mzML files using the msconvert module in Proteowizard. MzML files were converted to dia file format in DIA‐NN.^[^ ^60^ ^]^

### Library

A human canonical Swissprot fasta file was converted to a Prosit csv upload file with the convert tool in encyclopedia 0.9.0 (Searle 2018). The following settings were applied: Trypsin, up to 1 missed cleavage, range 396–1004 m/z, charge states 2+ and 3+, default charge state 3 and NCE 33. The csv file was uploaded to the Prosit webserver and converted to spectrum libraries in generic text format. The predicted human canonical library (20 374 protein isoforms, 28 325 protein groups and 1 626 266 precursors) was searched with the 6 GPF runs to generate a project specific library (12 280 protein isoforms, 13 383 protein groups and 150 809 precursors).

### Samples

625 sample files were searched with DIA‐NN 1.7.12 and the project library. The applied settings were: Output will be filtered at 0.01 FDR, N‐terminal methionine excision enabled, Maximum number of missed cleavages set to 1, Min peptide length set to 7, Max peptide length set to 30, Min precursor m/z set to 400, Max precursor m/z set to 1000, Cysteine carbamidomethylation enabled as a fixed modification, Scan window radius set to 10, Mass accuracy will be fixed to 3e‐05 (MS2) and 2e‐05 (MS1), Implicit protein grouping: protein names, Normalization: Rt dependend.

### ICP‐MS

Brain iron concentrations were measured in the biopsy tissue (≈50 mg) from the inferior temporal cortex from 625 post‐mortem brains. Tissue samples were cut into 50 ± 5 mg using a ceramic blade to avoid metal contamination. These specimens were weighed, then homogenised using Tris‐buffered saline (TBS) containing phosphatase and protease inhibitors. The tissue samples were stored at −80 °C until analysis. Iron levels from the brain tissues were measured using Inductively Coupled Plasma Mass Spectrometry (ICP‐MS; Ultramass 700, Varian). Tissue samples from each experimental condition were freeze‐dried, and then resuspended in 69% nitric acid (ultraclean grade, Aristar) overnight. The samples were then heated for 20 min at 90 °C, and an equivalent volume of hydrogen peroxide (30%, Merck) was added for a further 15 min incubation at 70 °C. The samples were diluted in double‐distilled water and assayed by ICP‐MS. Each tissue sample was measured in triplicate and the concentrations determined from the standard curve were normalized to wet tissue weight.

### Glutathione Measurement

Inferior temporal cortex tissue (10 mg) was homogenised in 100 µL Tris‐buffered saline containing phosphatase (Cat#P5726, Merck) and protease (Cat#05056489001, Merck) inhibitors and stored at −80 °C as previously described (Ref: Iron in the Alzheimer's brain: association with cognitive decline across multiple regions). 100 µL lysis buffer containing 2% IGE‐PAL (Cat#I3021, Merck) and butylated hydroxytoluene (Cat#ab118970, Abcam) was added and samples were incubated on ice for 30 min. Samples were centrifuged at 16 000 g for 10 min at 4 °C and supernatant collected. Protein concentrations were measured with a BCA based assay kit (Cat#23225, Thermo Fisher Scientific). Total intracellular GSH was measured using a modified monochlorobimane (mCB) method previously described.^[^ ^26^ ^]^ In brief, 50 µL of 2x mCB working reagent composed of equine spleen glutathione S‐transferase (3 U mL^−1^, mCB (300 µm) in HBSS additionally containing, 1 mm sodium bicarbonate, HEPES (20 mm), pH 7.4, 10 mm d‐glucose was added to 50 µL of sample supernatant and samples shaken at 450 rpm for 30 min. Fluorescence (excitation: 394 nm, emission: 490 nm) was measured using a microplate reader (Clariostar, BMG Labtech). Total GSH levels were expressed relative to protein concentration.

### Cystine Measurement

The cystine variable was part of a single batch metabolomics data set of 150 metabolites from 323 participants received on 29/02/2024 derived by Metabolomics Australia. The data was checked for extreme values and drift corrected using the pmp() package.^[^ ^61^ ^]^ Exploration of the data showed that the PBQC samples followed a different and more extreme drift pattern compared to the sample data and was deemed inappropriate to model drift for the purposes of drift correction. Instead, the sample data was used.

### Cell Culture and Reagents

HT22 cells, derived from mouse hippocampus (Cat#SCC129, Merck), were cultured in RPMI 1640 growth media (Cat#72400120, Thermo Fisher Scientific) supplemented with 10% (v/v) FBS (Bovogen biologicals), penicillin and streptomycin (Cat#15140122, Thermo Fisher Scientific). Cells were seeded and treated in DMEM (Cat# A1443001, Thermo Fisher Scientific) supplemented with 10% (v/v) FBS and penicillin and streptomycin as well as 20 mm HEPES, pH 7.4 (Cat#15630080, Thermo Fisher Scientific) and 5 mm d‐glucose (Cat#G7528, Merck). All cells were cultured at 37 °C with 5% CO_2_.

Erastin (Cat#S7242) and RSL3 (Cat#S8155) were purchased from Selleckchem. BSO (Cat#B251), arachidonic acid (Cat#10931) and ammonium iron(II) sulfate hexahydrate (Cat#203505), liproxstatin‐1 (LPX, Cat#SML1414), glutathione ethyl ester (Cat# G1404) were purchased from Merck. The electron transport chain inhibitors used were: Rotenone (Cat# R8875, Merck), TTFA (Cat# ab223880, Abcam), antimycin A (Cat# A8674, Merck), sodium azide (Cat#438456, Merck) and CCCP (Cat#C2759, Merck).

HT‐22 rho⁰ (ρ⁰) and parental rho⁺ (ρ⁺) mouse hippocampal neuronal cell lines were obtained from Kerafast, Inc. (Boston, MA, USA). The ρ⁰ cells were originally generated by Kozhukhar and Alexeyev (2024)^[^ ^62^ ^]^ through combined treatment of wild‐type HT‐22 cells with 200 µm 2′, 3′‐dideoxycytidine (ddC) and 0.5 µg mL^−1^ ethidium bromide (EtBr), followed by clonal isolation and confirmation of mitochondrial DNA (mtDNA) loss via duplex PCR analysis.

ρ⁰ cells were maintained in high‐glucose Dulbecco's Modified Eagle Medium (DMEM; 4.5 g L^−1^ glucose) supplemented with 1 mm sodium pyruvate and 50 µg mL^−1^ uridine to support growth in the absence of mtDNA‐encoded respiratory function. Parental ρ⁺ HT‐22 cells were cultured in the same DMEM formulation without supplementation. All media were supplemented with 10% fetal bovine serum (FBS), and cultures were maintained at 37 °C in a humidified incubator with 5% CO₂.

For subculturing, ρ⁺ cells were passaged at a 1:20 ratio every 3 days, while ρ⁰ cells were passaged at a 1:3 ratio due to their slower proliferation rate.

### Cell Viability Assay

Cell viability was evaluated using the calcein‐AM assay (Cat#C025, Sapphire Bioscience)). Briefly, cells were cultured in 96‐well plate at a density of 20 000 cells/well in treatment media for 16 h. Cells were subsequently incubated with candidate compounds in treatment media for an additional 17 h. Cells were washed with HBSS containing 20 mm HEPES, pH 7.4, 10 mm d‐glucose, and then calcein‐AM (1 µm) dissolved in the same buffer was added, incubated for 30 min at 37 °C and fluorescence (excitation: 485 nm, emission: 530 nm) was measured using a microplate reader (Clariostar, BMG Labtech).

### ATP Assay

Intracellular ATP was determined by CellTiter‐Glo luminescence kit (Promega, G7570), which can perform cell lysis and generate a luminescent signal proportional to the amount of ATP present. In brief, cells were washed with HBSS before 100 µL of CellTiter‐Glo luminescence test solution was added and incubated for 10 min at room temperature. Luminescence was measured using a microplate reader (Clariostar, BMG Labtech).

### Liposomal ATP Treatment

Liposome preparations (Encapsula NanoSciences, Nashville, TN, USA) were composed of phosphatidylserine, phosphatidylchonline, 1,2‐dioleoyl‐sn‐glycero‐3‐phosphoethanolamine‐N‐(7‐nitro‐2‐1,3‐benzoxadiazol‐4‐yl) (ammonium salt) (NBD PE) as a molar ratio percentage of 30:69:1. The ATP content of ATP‐liposomes was determined for each batch before use. For ATP replenishment experiments, following 24 h in culture, the medium was replaced with fresh medium containing 5% of either ATP‐encapsulated or empty liposomes, continuing the co‐incubation with mitochondrial inhibitors ((antimycin A – Complex III inhibitor (250 nm); sodium azide – Complex IV inhibitor (7.5 mm); CCCP – mitochondrial uncoupler (3.125 µm)) for 17 h. After this treatment, cells were washed thoroughly with PBS and collected for ATP analysis.

### GSH Assays

Total intracellular GSH was measured using a modification of a previously described monochlorobimane (mCB) assay.^[^ ^63^ ^]^ In brief, cells were washed with supplemented HBSS as for the “Cell viability vssay” before 100 µL of mCB working reagent as above, except containing glutathione S‐transferase (0.5 U mL^−1^) and mCB (30 µm), shaken at 450 rpm for 2 min, and incubated for 60 min at 37 °C. Fluorescence (excitation: 394 nm, emission: 490 nm) l was measured using a microplate reader (Clariostar, BMG Labtech).

GSH/GSSG ratios were measured using a luminescent‐based assay to detect and quantify total glutathione ratios GSH/GSSG‐Glo Assay (Promega, V6611). The experiments were performed according to previously described methods.^[^ ^64^ ^]^

### Western Blot Analysis

Proteins were extracted from cell pellets using RIPA buffer (50 mm Tris, 150 mm NaCl, 0.1% SDS, 0.5% sodium deoxycholate, 1% Triton X‐100, containing protease inhibitors (Cat#05056489001, Merck)). Protein concentrations were measured with a BCA based assay kit (Cat#23225, Thermo Fisher Scientific). 15 µg aliquots of protein were separated by on 4–20% Bis‐Tris protein gels (Cat#WG1403BOX, Thermo Fisher Scientific) and transferred to polyvinylidene difluoride membrane (Cat# IB24001, Thermo Fisher Scientific). Primary antibodies used were SLC25A39 (1:100, cat# 14963‐1‐AP, ProteinTech) and anti‐β‐actin (cat#A5441, Merck). Membranes were probed with horseradish peroxidase‐conjugated secondary antibodies (Cat#P044801‐2 and P044701‐2, Elitech) and signal was detected using a LAS‐4000 analyzer (GE Healthcare Life science). Densitometry analyses were carried out using Image J^[^ ^65^ ^]^ and quantitation was normalized to β‐actin levels.

### Statistical Analysis—Cell culture

All cell culture data were analyzed using Prism 9.0.1 (Graphpad). Replicates within experiments are indicated in each figure legend and correspond to biological replicates and three independent experiments were used to ensure reliability of the reported values. Data were expressed as mean ± standard error of the mean (SEM) from independent experiments. One‐way ANOVA was used to compare between groups and calculate the *p*‐value with Benjamini–Krieger–Yekutieli (BKY) correction as indicated in each figure legend. A two way *p*‐value at α < 0.05 was considered statistically significant. For the cell viability data, non‐linear regression analysis with a variable slope model was used to fit a curve to dose response.

### Data Analysis

Identifier columns and MaxLFQ intensities were extracted from the DIA‐NN output file and converted to a matrix format using an R script (R version 4.4.2). Data were imported into Perseus^[^ ^66^ ^]^ for further statistical analysis. Given the exploratory nature of this analysis *p* values have not been adjusted for multiple testing.

### Data Imputation and Batch Correction

An initial look at the gene data revealed a pattern of change based on data processing batch and required batch correction. Batch correction was conducted using the proBatch package. Data was log2 transformed and quantile normalised. To correct for within‐batch variation (drift), loess fitting was used. Discrete batch effects (between batch) was corrected using gene‐level median centering. Two datasets were generated for the *n* = 670 subjects, an imputed dataset and an unimputed dataset. Imputation process required the removal of any genes that had < 70% data therefore, 13 genes were removed, and 3098 genes remained. Hot deck imputation using the VIM package^[^ ^67^ ^]^ using age of death as an order variable and sex as a domain variable was used to generate data for these genes. The unimputed data set used all 3111 genes with a total of 97 261 NA observations (4.66% missingness).

Subjects were grouped into three groups representing their clinical and pathological status based on the Consortium to Establish a Registry for Alzheimer's Disease (CERAD) criteria. The groups (DX group) were: people who died without dementia and were classified negative for AD pathology (based on the Consortium to Establish a Registry for Alzheimer's Disease [CERAD] criteria; ND‐ve; *n* = 165), people who died without dementia but were classified as positive for AD pathology (ND+ve; *n* = 213) and people who were diagnosed with AD during their life, and had AD pathology confirmed post mortem (AD+ve *n* = 237).

### Weighted Gene Co‐Expression Network Analysis (WGNCA)

To explore clusters of correlation patterns among genes across samples. A weighted gene co‐expression network analysis (WGCNA) was run. Genes were clustered into modules using the WGCNA package in Ref.[18] The data was processed, and outliers were removed guided by sample clustering. One‐step network construction and module detection were run with power = 7 and an unsigned topological overlap matrix (TOMType) A cluster dendrogram showed 7 clear module clusters. Next, the modules were filtered for hub genes. These are genes within the co‐expression modules that are highly connected with each gene in the module based on correlation with eigengenes. These hub genes were then modeled against external clinical traits. Investigation of the genes associated with module yellow (MEyellow) identified MEyellow as a mitochondrial hub gene and used in further linear analyses. The WGCNA was replicated on a separate cohort,^[^ ^19^ ^]^ n proteins = 3331, n subject = 479, with a power of 4 and to reveal 9 modules.

### Longitudinal Linear Mixed Model Building

To test the pattern of change in longitudinal cognition between high and low GSH and m29 composite scores linear mixed effects models were run. Longitudinal cognition scores across Episodic memory, Working memory, Semantic memory, visuospatial ability, perceptual speed, and a global cognition composite. Effects on cognition were modeled as the interaction between the diagnosis group and time (years prior to death) with age at death, sex and a binary E4 variable (1 if any e4 allele was present, 0 otherwise) as covariates. The models included a person‐specific random level term with an unstructured variance‐covariance matrix. Degrees of freedom for the purposes of generating p values were approximated using Satterthwaite's method.

### Total GSH Over Pathological and Clinical Diagnosis

To explore the expression of total GSH with any given protein across clinical and pathological groupings, the outcome total GSH was modeled with the interaction between DX group and each gene available in our data set with age at baseline, sex and a binary E4 variable as covariates.

Using the genes identified in the linear model, differences in patterns of correlation between total GSH and a collection of 162 genes (29 of which were enriched in cellular component GO:0005743) within DX group were explored. These genes were identified as statistically significant in the earlier linear models.

### Differential Protein Expression

Differences between groups was calculated on the unimputed batch corrected peptides (*n* = 3111). Log10 p‐values and log2 fold change were used to create volcano plots and statistically significant peptides were exported and presented as volcano plots, which were generated with the ggplot2 package in R.

### Pathway Enrichment

To characterize co‐expressed proteins based on GO annotation,^[^ ^68^ ^]^ enrichment lists for Reactome, Kegg, Biological Process, cellular component and molecular function were generated using DAVID GO. DavidGo out‐put was input into monaGO for chord plot visualization.^[^ ^69^ ^]^

## Conflict of Interest

The authors declare no conflict of interest.

## Author Contributions

SA and FA conceptualized the project. FA, DL, and PK designed the experiments. FA, DL, MJ, PK, AS, AAB, TSE, TPMN, PL, MK, and SM conducted the experiments and analyses. PA, SEL, and JS curated and analyzed the human data. FA, DL, AW, PL, MC, PA, SEL, JS, AIB, and SA provided conceptual input. FA, DL, AW, PK, and SA drafted the manuscript, and FA, DL, AW, PK, AAB, PL, MC, PA, SEL, JS, AIB, and SA edited it. SA, AB, and JS secured funding for the study. All authors read and approved the final manuscript.

## Supporting information



Supporting Information

## Data Availability

The data that support the findings of this study are available from the corresponding author upon reasonable request.
