# Supplementary material for: Aberrant Mitochondrial Metabolism in Alzheimer's Disease Links Energy Stress with Ferroptosis
Source: Adv Sci (Weinh). 2025 Jul 8;12(37):e04175. doi: 10.1002/advs.202504175 (PMC12499490; doi:10.1002/advs.202504175)
Supplement: Supplementary file 1 — Supporting Information [file ADVS-12-e04175-s001.docx]

Supporting Information

Aberrant mitochondrial metabolism in Alzheimer's disease links energy stress with ferroptosis

*Francesca Alves^1,2^, Darius Lane^1^, Adam Wahida^3^, Md. Jakaria^1^, Pawel Kalinowski^1^, Adam Southon^1^, Abdel Ali Belaidi^1^, Triet Phu Minh Nguyen^1^, Peng Lei^4^, Marcus Krueger^5,6^, Stefan Mueller^7^, Marcus Conrad^3^*^,8^*, Puja Agarwal^9^, Sue E Leurgans^9^, Julie Schneider^9^, Ashley I. Bush^1,2^, Scott Ayton^1,2,*^*


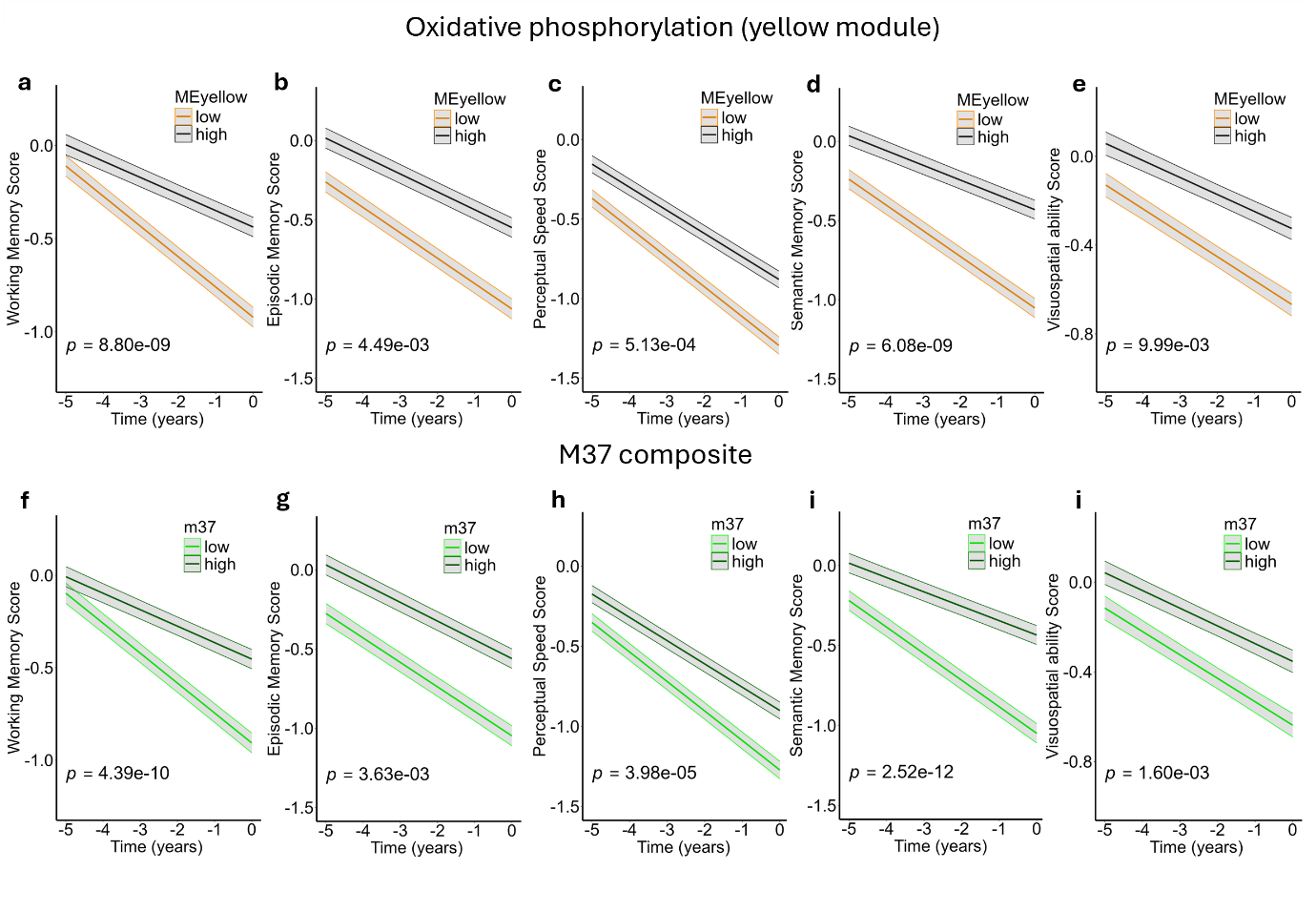


**Figure S1: Low MEyellow(oxidative/mitochondrial module) is associated with accelerated cognitive decline in a battery of cognitive tests.** Longitudinal cognition scores across **a,** Working memory, **b,** Episodic memory, **c,** perceptual speed, **d,** Semantic memory and **e,** visuospatial ability were modelled and grouped according to (median) high or low MEyellow eigenvalue or **f-j** high or low mitochondrial composite of 29 proteins (m29) with age at death, sex and e4 covariates (subjects n=615). Degrees of freedom for the purposes of generating p values were approximated using Satterthwaite’s method. Error bars represent 95% CIs.


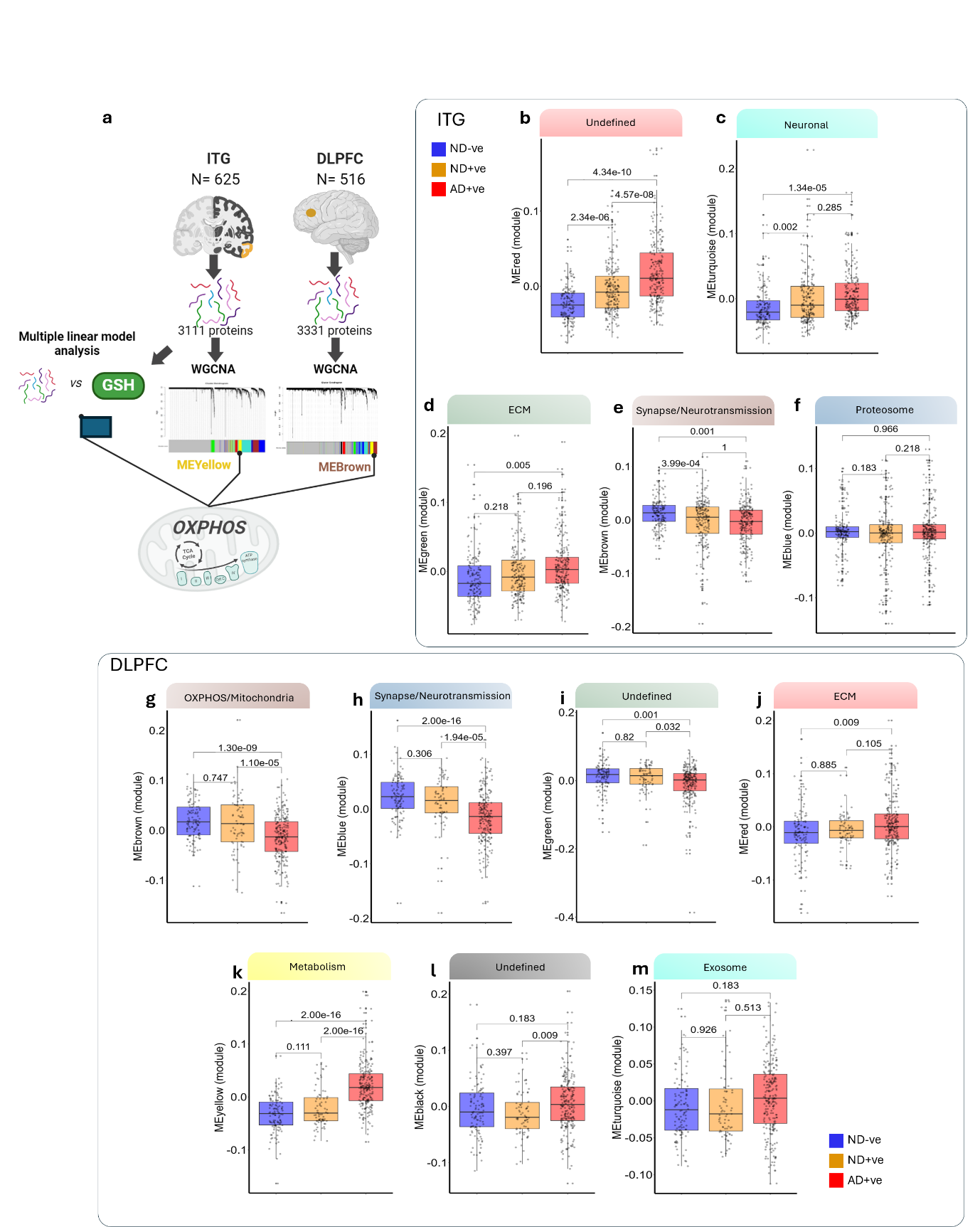


**Figure S2: WGCNA of validation dataset from DLFP confers mitochondrial/oxphos module. a,** schematic diagram of parallel WGCNA analysis in the RUSH cohort using inferior temporal gyrus (ITG) and validation data set from Johnson (2022) using dorsolateral prefrontal cortex (DLFP), created using BioRender.com. **b-f**, boxplots of eigenvalues from assigned modules from the RUSH cohort) N= 625 and **g-m**, Johnson (2022)^[19]^ cohort N = 516. Each dot represents individual subject; Rush data was analysed using ANCOVA with age at death sex and E4 covariates Johnson data were analysed with ANOVA.


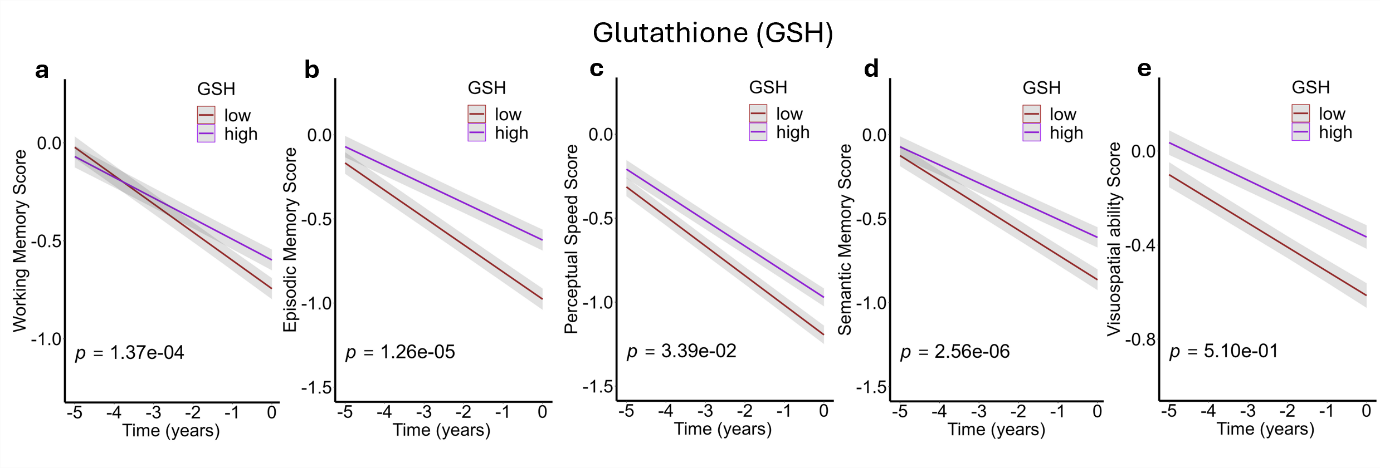


**Figure S3: Low GSH is associated with accelerated cognitive decline in a battery of cognitive tests.** Longitudinal cognition scores across **a,** Working memory, **b,** Episodic memory, **c,** perceptual speed, **d,** Semantic memory and **e,** visuospatial ability were modelled and grouped according to high or low GSH with age at death, sex and e4 covariates (subjects n=615). Degrees of freedom for the purposes of generating p values were approximated using Satterthwaite’s method.


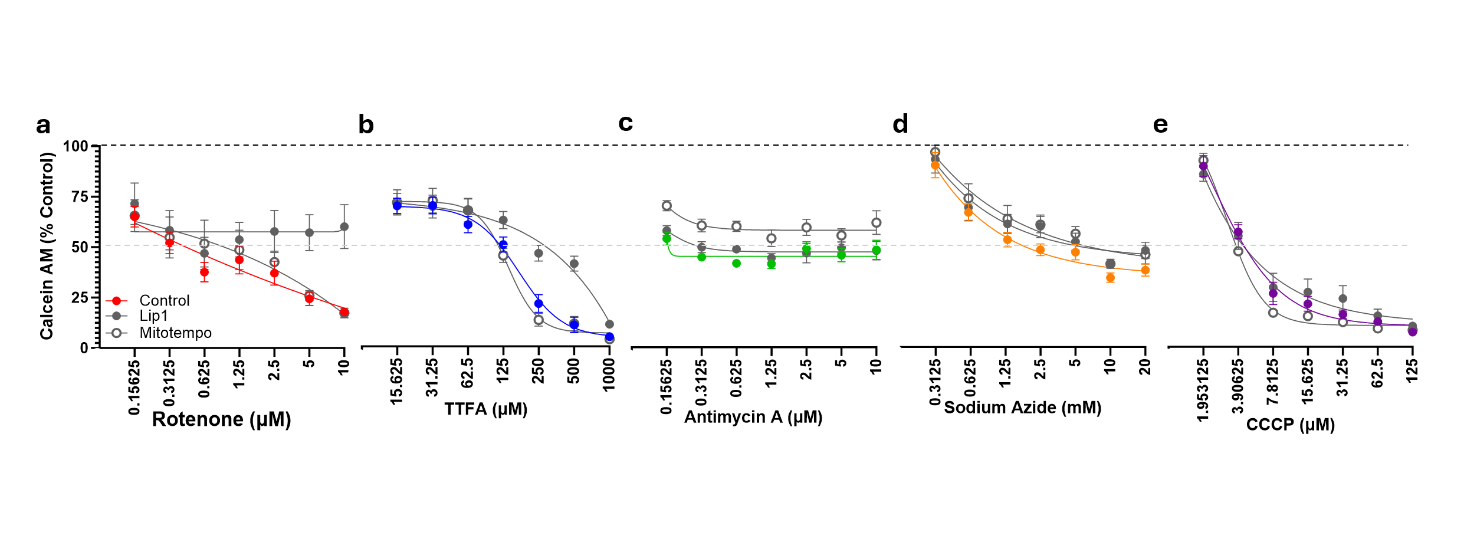


**Figure S4: Exclusion of ETC inhibitors that cause ferroptosis.** Viability assays of HT22 cells co-treated with OXPHOS inhibitors a, Rotenone; b, TTFA; c, Antimycin A; d, Sodium Azide and e, CCCP in the presence or absence of liproxstatin (lip1) and Mitotempo. Data presented as mean+/-SEM from 3 independent experiments.

**Figure S5: Glutathione synthesis inhibition does not increase ATP.** ATP was measured after 17 hour treatment with/without BSO (100μM) in the presence of liproxstatin (LPX). Data presented as mean+/-SEM from 3 independent experiments.


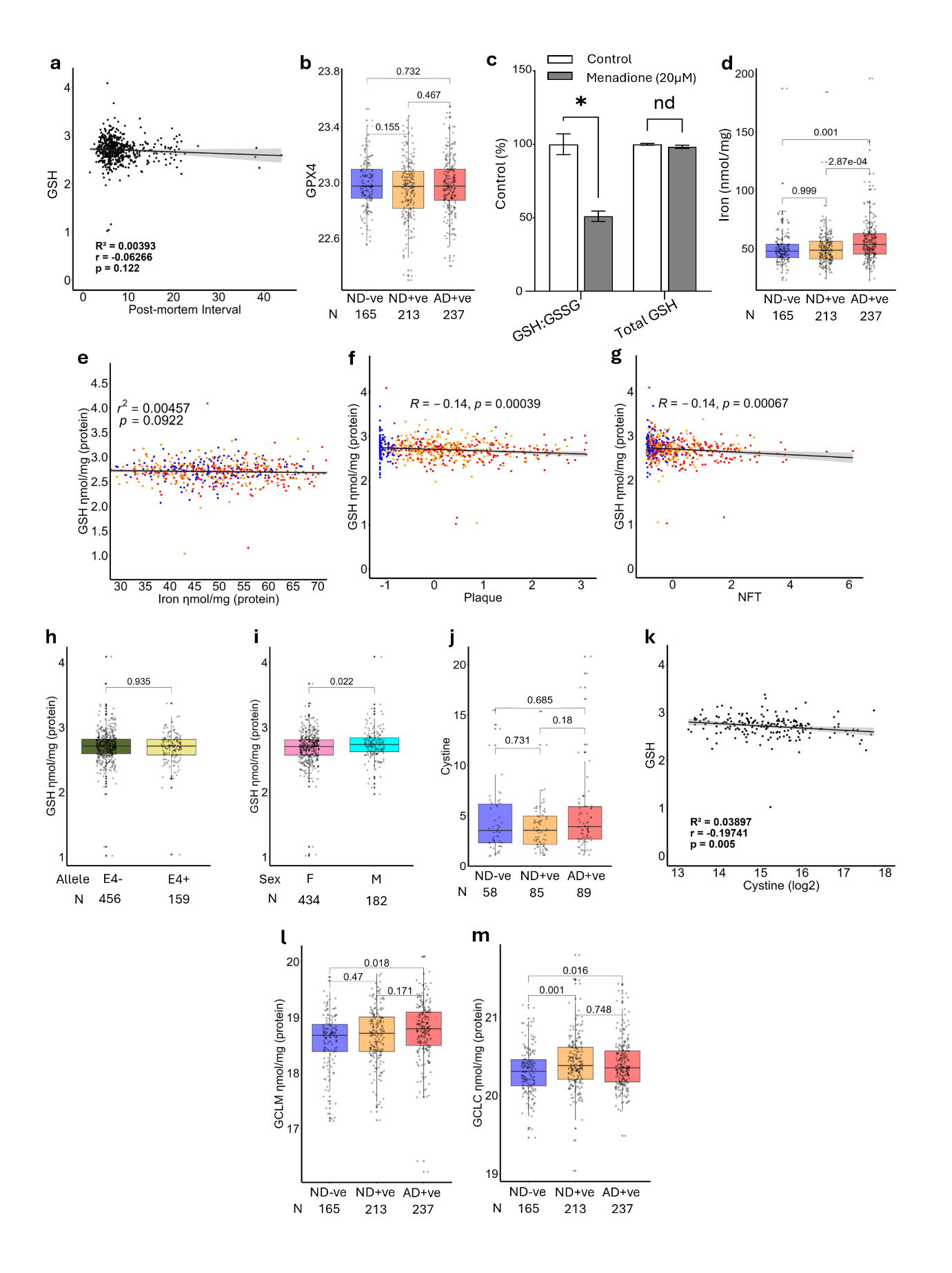


**Figure S6: Alternative explanations for low glutathione in Alzheimer’s disease are not valid. a,** correlation between GSH and post mortem interval N=625. **b**, Boxplot of glutathione peroxidase 4 (GPX4) across ROSMAP study subjects assigned according to dementia and pathology (CERAD), **c**, GSH:GSSG ratio and total GSH from HT22 cells treated for 1 hour with (20uM) menadione to induce oxidative stress; data presented as mean+/-SEM of 3 independent experiments, multiple t-tests **d**, Boxplot of iron levels from ICP-MS and **e**, correlation of iron and GSH levels, **f**, GSH and plaque and **g**, GSH and neurofibrillary tangles (NFT) ; each dot represents a single participant (AD+ve = red, ND+ve = yellow, ND-ve = blue), shading represent 95% CIs. Boxplots of GSH abundance according to **h,** APOE e4 status and **i,** sex. **j,** Boxplot of cysteine levels (relative abundance) and **k,** correlation between GSH and cystine (log2). Boxplots of gamma-glutamylcysteine synthetase subunits **l,** Glutamate-Cysteine Ligase Modifier Subunit (GCLM) and **m,** Glutamate-Cysteine Ligase Catalytic Subunit
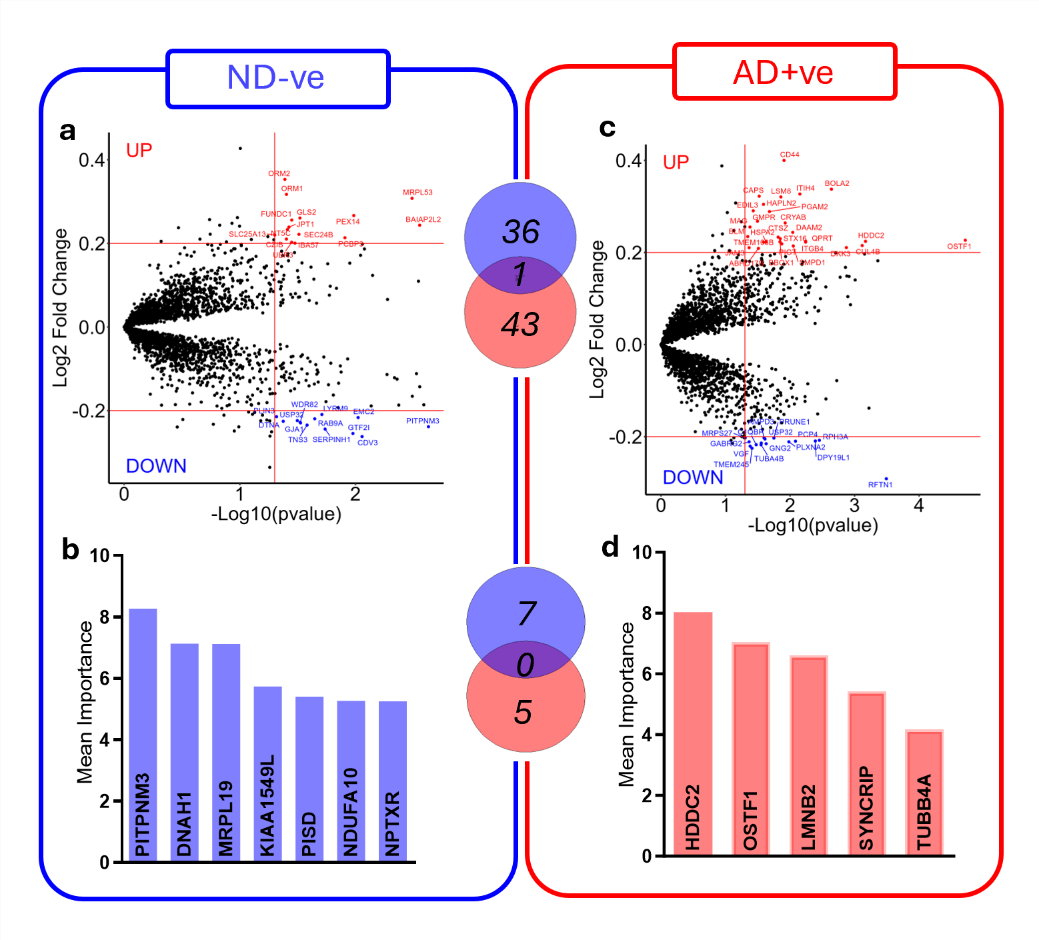
(GCLC).

**Figure S7: No common biochemical pathway associated with GSH in control-ve and AD+. a,** Differential protein expression and **b,** Boruta analysis in ND-ve to predict High vs Low GSH (based on a median centre split). **c,** Differential protein expression and **b,** Boruta analysis in AD+ve to predict High vs Low GSH (based on a median centre split). Venn diagrams display common significant hits between ND-ve (blue) and AD+ (red).


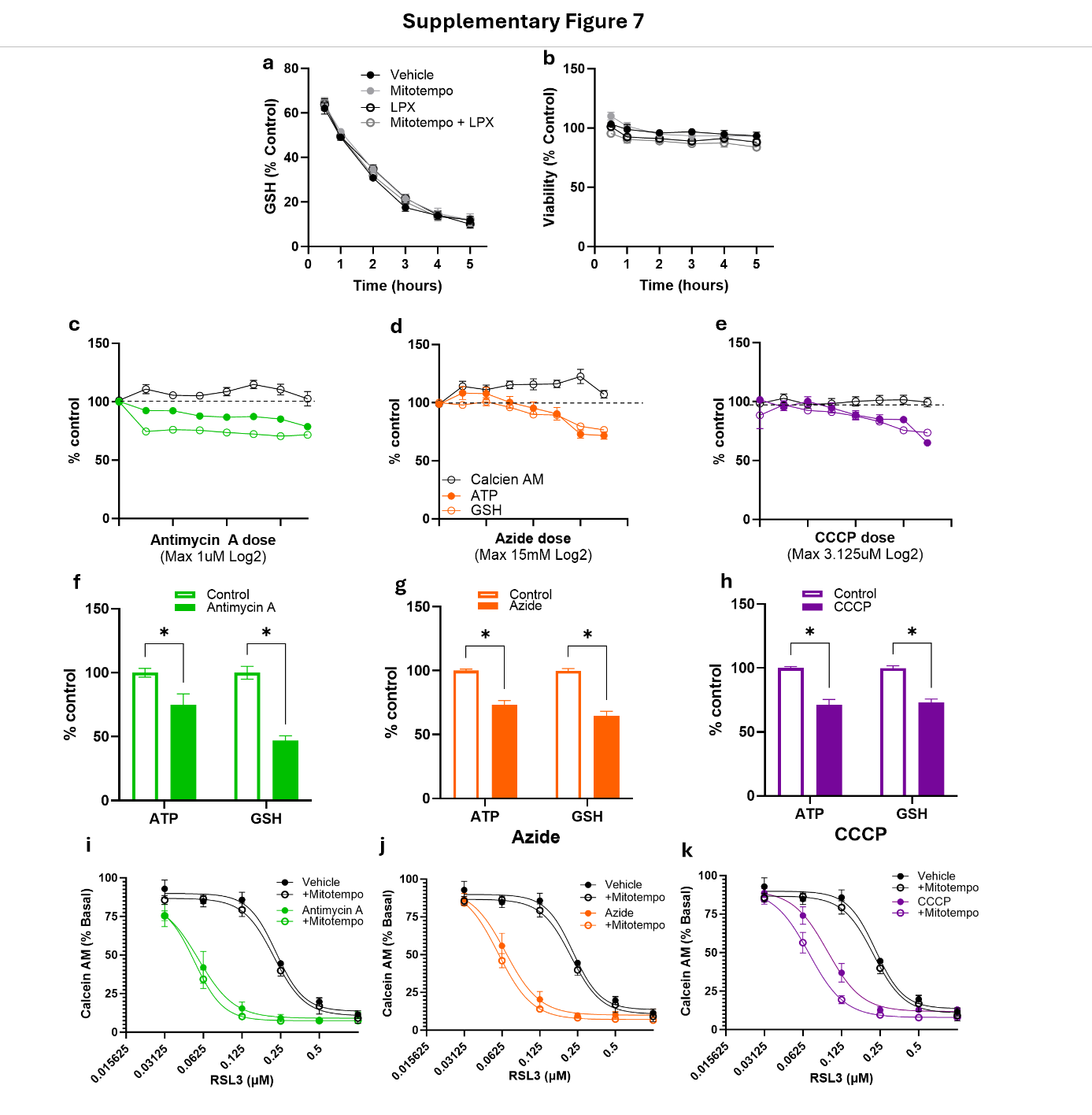


**Figure S8: Glutathione consumption or production is not altered by mitochondrial oxidative stress scavenger mitotempo. a**, Total GSH and **b**, viability cells (assayed by Calcein AM) every hour during a 5-hour incubation of HT22 cells with Erastin (10uM) in the presence or absence of mitotempo and/or liproxstatin-1. **c-e,** 5 Hour incubation and **f-h**, Total GSH and ATP levels after 17 hour incubation of HT22 neuronal co-treated with electron transport chain inhibitors (c, antimycin A – Complex III inhibitor (green, 200nM); sodium azide – Complex IV inhibitor (orange, 7.5mM); CCCP – mitochondrial uncoupler (purple, 3.125μM)). **i-j,** ATP and GSH of HT22 neuronal cells (assayed by Calcein AM) co-treated with RSL3 for 17 hours with electron transport chain inhibitors (**f,** antimycin A –200nM; **g,** sodium azide –7.5mM; **h,** CCCP –3.125μM)) in the presence or absence of mitotempo (1μM). Data presented as mean+/- SEM of 3 independent experiments.

**
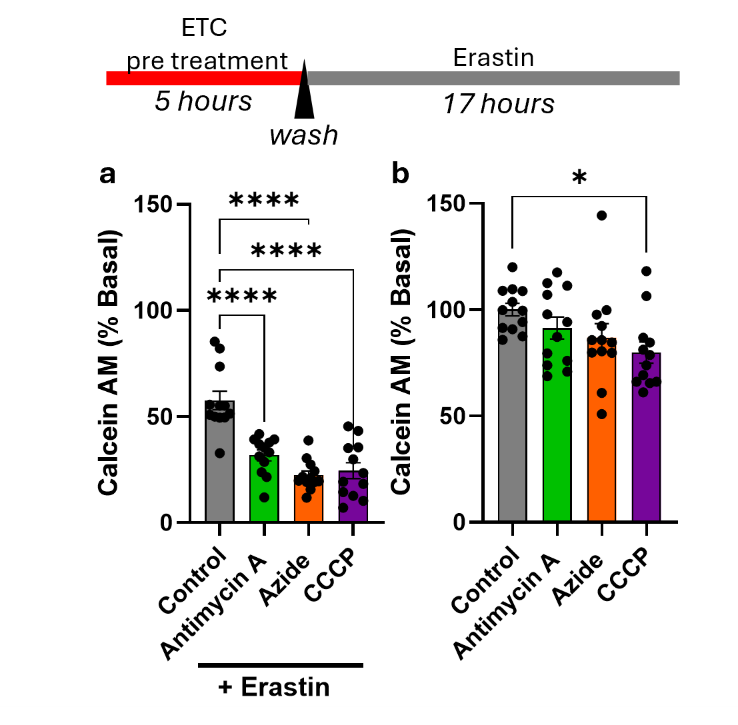
**

**Figure S9: Electron transport chain inhibitor pretreatment renders cells more vulnerable to erastin induced toxicity. a**, viability of cells (assayed by Calcein AM) after a 5-hour incubation of HT22 cells with/without electron transport chain inhibitors (c, antimycin A – Complex III inhibitor (green, 200nM); sodium azide – Complex IV inhibitor (orange, 7.5mM); CCCP – mitochondrial uncoupler (purple, 3.125μM)) followed by a wash and 17 hour treatment with Erastin (20uM). **b**, basal viability without erastin treatment. one way ANOVA, **** p<0.0001, *p<0.05. Data presented as mean+/- SEM of 3 independent experiments.

**
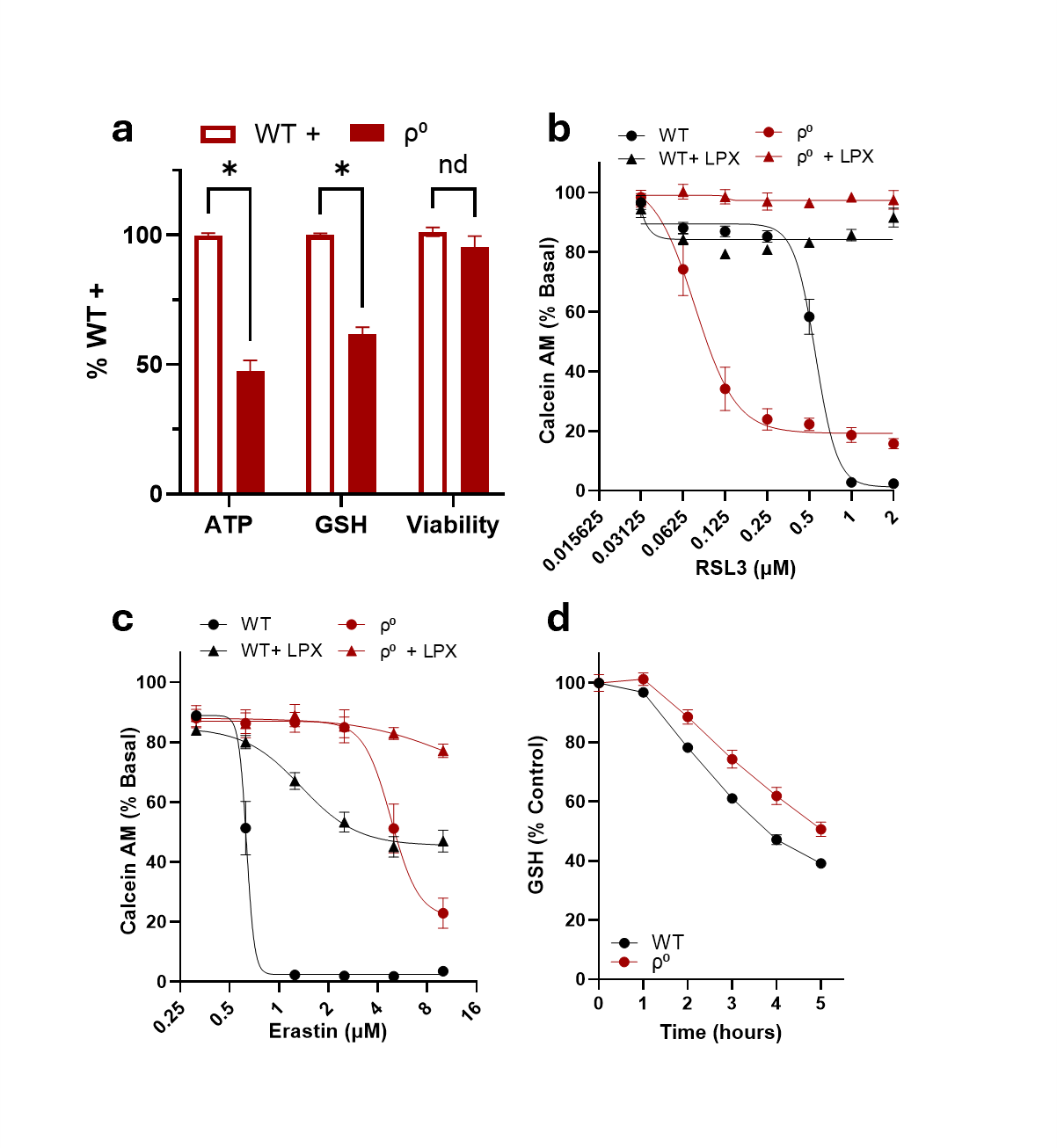
**

**Figure S10: Rho nought (ρ⁰) cells exemplify how mitochondria act as both a producer and consumer of glutathione depending on the context. a,** bar plots of ATP, t-GSH and viability (calcien AM) in HT22 ρ⁰ and wild type (WT+) cells. viability curves of b, RSL3 and **c,** erastin. **d,** total GSH depletion with erastin (10μM) in WT (black) and ρ⁰ (red) cells. Data presented as mean+/- SEM of 3 independent experiments.


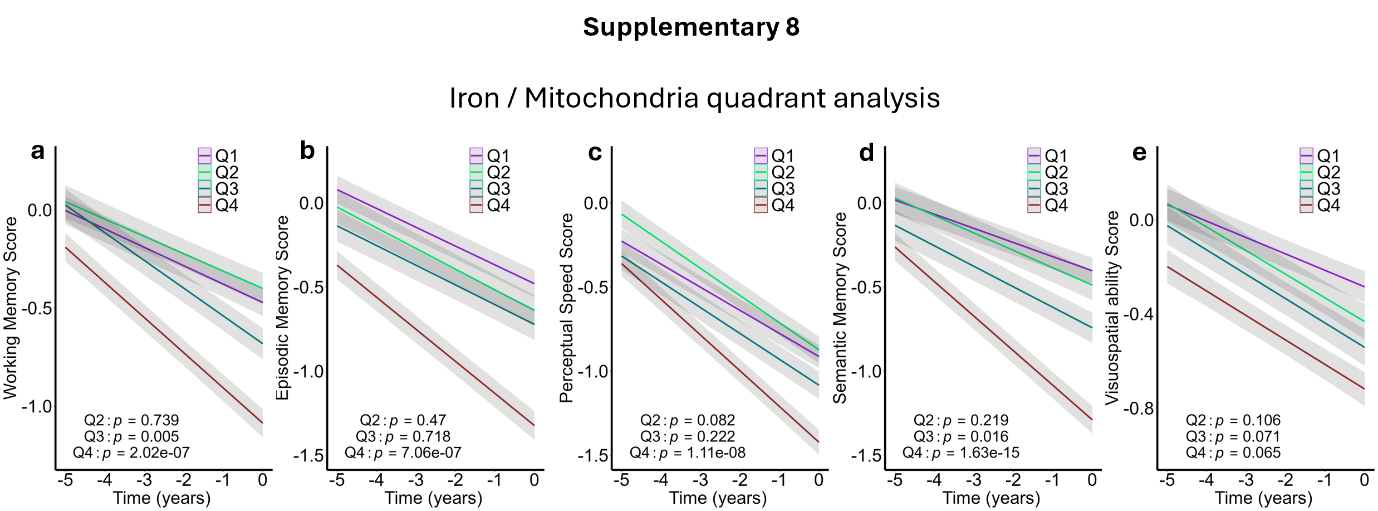


**Figure S11: Q4 is associated with accelerated cognitive decline in a battery of cognitive tests.** Longitudinal cognition scores across **a,** Working memory, **b,** Episodic memory, **c,** perceptual speed, **d,** Semantic memory and **e,** visuospatial ability were modelled and grouped according to quadrant assignment (all subjects with longitudinal cognition; n=615). Statistics from linear mixed model using quadrant vs time interaction and age at death sex and e4 as covariates. Degrees of freedom for the purposes of generating p values were approximated using Satterthwaite’s method. Error bars represent 95% CIs.
